# Sustained elevation of MG53 in the bloodstream increases tissue regenerative capacity without compromising metabolic function

**DOI:** 10.1038/s41467-019-12483-0

**Published:** 2019-10-11

**Authors:** Zehua Bian, Qiang Wang, Xinyu Zhou, Tao Tan, Ki Ho Park, H. Fritz Kramer, Alan McDougal, Nicholas J. Laping, Sanjay Kumar, T. M. Ayodele Adesanya, Matthew Sermersheim, Frank Yi, Xinxin Wang, Junwei Wu, Kristyn Gumpper, Qiwei Jiang, Duofen He, Pei-Hui Lin, Haichang Li, Fangxia Guan, Jingsong Zhou, Mark J. Kohr, Chunyu Zeng, Hua Zhu, Jianjie Ma

**Affiliations:** 1 Department of Surgery, Davis Heart and Lung Research Institute, The Ohio State University, Columbus, OH 43210 USA; 2 Innate Immunity Research Unit, GlaxoSmithKline, Inc., Collegeville, PA 19426 USA; 3 Novel Human Genetics Research Unit, GlaxoSmithKline, Inc., Collegeville, PA 19426 USA; 4 The First Affiliated Hospital of Zhengzhou University, Zhengzhou, Henan 45000 China; 5 Department of Cardiology, Daping Hospital, The Third Military Medical University, Chongqing, 400042 China; 6 School of Life Sciences, Zhengzhou University, Zhengzhou, Henan 450001 China; 7 College of Nursing and Health Innovation, University of Texas at Arlington, Arlington, TX 76019 USA; 8 Department of Environmental Health and Engineering, Bloomberg School of Public Health, Johns Hopkins University, Baltimore, MD 21205 USA

**Keywords:** Cell biology, Molecular biology, Physiology, Systems biology, Molecular medicine

## Abstract

MG53 is a muscle-specific TRIM-family protein that presides over the cell membrane repair response. Here, we show that MG53 present in blood circulation acts as a myokine to facilitate tissue injury-repair and regeneration. Transgenic mice with sustained elevation of MG53 in the bloodstream (tPA-MG53) have a healthier and longer life-span when compared with littermate wild type mice. The tPA-MG53 mice show normal glucose handling and insulin signaling in skeletal muscle, and sustained elevation of MG53 in the bloodstream does not have a deleterious impact on db/db mice. More importantly, the tPA-MG53 mice display remarkable dermal wound healing capacity, enhanced muscle performance, and improved injury-repair and regeneration. Recombinant human MG53 protein protects against eccentric contraction-induced acute and chronic muscle injury in mice. Our findings highlight the myokine function of MG53 in tissue protection and present MG53 as an attractive biological reagent for regenerative medicine without interference with glucose handling in the body.

## Abstract (web summary)

MG53 is a protein that regulates the cell membrane repair process, and it’s been suggested that it might play a role in diabetes. Here, the authors demonstrate that circulating MG53 functions as a myokine to facilitate tissue injury-repair and regeneration without impacting glucose handling.

## Introduction

Skeletal muscle controls body locomotion and requires an active injury-repair mechanism to maintain its integrity, as contraction-relaxation of muscle fibers often causes injury to the sarcolemma. Developing therapeutic approaches to improve sarcolemma integrity and facilitate regeneration of injured muscle fibers remains a major challenge in muscle physiology research^1–3^. In addition to being a contractile machine, skeletal muscle is recognized as an endocrine organ, secreting a myriad of myokines to modulate other tissue functions^4–8^. Targeting the myokine function of skeletal muscle and its cross-talk with other tissues is an attractive means to boost the body’s regenerative capacity under physiological and pathological conditions.

MG53, a member of the tripartite motif (TRIM) family proteins, plays an essential role in plasma membrane damage repair^9–11^. MG53 null mice display progressive skeletal myopathy and decreased regenerative capacity of the cardiomyocytes due to compromised cell membrane repair function^9,10,12^. MG53 is also found in blood circulation at levels correlating with muscle contractile and secretory activities^13–16^. Extracellular application of the recombinant human MG53 (rhMG53) protein protects various types of cells against membrane disruption. When applied intravenously, rhMG53 ameliorates pathology associated with muscular dystrophy, lung injury, myocardial infarction, and acute kidney injury in rodent and large animal models of these diseases^13,17–19^. These data highlight the importance of targeting cell membrane repair in regenerative medicine and present MG53 as a potential biological reagent for restoration of tissue integrity in a broad range of human diseases.

In addition to cell membrane repair, MG53 contains the conserved RING motif with E3-ligase activity that can regulate degradation of its substrate via ubiquitin proteasome pathway. Song et al.^20^ reported that MG53 protein was increased in animal models of diabetes and proposed that MG53 functions as an E3-ligase to downregulate insulin receptor substrate 1 (IRS-1), serving as a causative factor for the development of diabetes. However, Yi et al.^21^ and other investigators^16,22,23^ observed normal MG53 expression in muscle samples of diabetic murine models and human patients. Moreover, early studies with knockout of IRS-1 in mice revealed no clear phenotype of type II diabetes^24,25^ as the existence of other IRS isoforms may compensate for IRS-1 absence^26–29^. Therefore, the proposed role for MG53 mediated IRS-1 downregulation in the manifestation of diabetes lacks a biological base. A recent report by Liu et al. showed that cardiac-specific overexpression of MG53 induced cardiomyopathy via transcriptional activation of the peroxisome proliferation-activated receptor (PPARα)^30^. While these studies raise concerns over the safety of MG53 overexpression on cardiovascular and metabolic function, no studies have been conducted to investigate the physiological impact of elevating MG53 in blood circulation.

We develop a transgenic mouse model with sustained elevation of MG53 in the bloodstream in order to examine the myokine function of MG53 in tissue injury-repair and regeneration. We observe that the tPA-MG53 mice live a healthy life-span and display enhanced tissue regenerative capacity. Physiological and biochemical studies reveal that sustained elevation of MG53 in the bloodstream does not impact the body’s metabolic function of glucose handling and insulin signaling.

## Results

### Mice with elevation of circulating MG53 are healthy

**Fig. 1:**
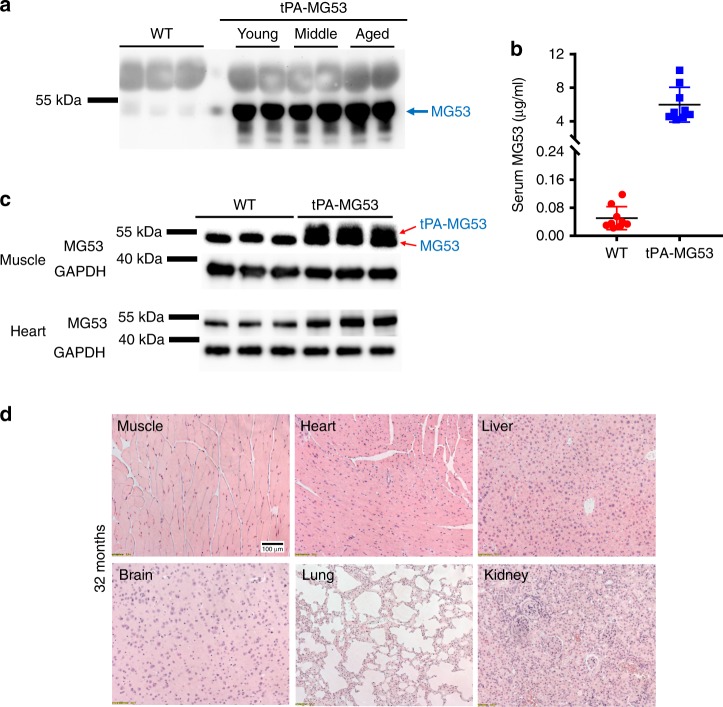
Mouse model with sustained elevation of MG53 in the bloodstream. **a** 1 µl sera derived from 3-month wild type (WT) and tPA-MG53 mice at 2 months (young), 12 months (middle) and 24 months (aged) were probed with anti-MG53 antibody. **b** Quantification of serum levels of MG53 in wild type and tPA-MG53 mice by western blot (*n* = 10, wild type; *n* = 9 for tPA-MG53, *P* < 0.0001). **c** Skeletal muscle (2 µg per lane) and heart (2 µg per lane) derived from tPA-MG53 and WT littermate mice were blotted with anti-MG53 antibody. Red arrows indicate doublet of MG53 and tPA-MG53. **d** H&E staining of vital organs from 32-month-old tPA-MG53 mice show normal tissue morphology. The pictures are representative of two other tPA-MG53 mice at 32 months age. Error bar represents the standard deviation and *P* value was generated by *t* test

In rodents, MG53 is abundantly expressed in skeletal and cardiac muscle tissues. Low levels of MG53 can be detected in the bloodstream under normal conditions^13,14,16^. To test the myokine function of MG53 in tissue repair and regeneration, we generated a transgenic mouse model with sustained elevation of MG53 in blood circulation (tPA-MG53) (Fig. 1). To promote the secretion of MG53 into blood circulation, a tissue plasminogen activator (tPA) sequence was added ahead of the human *mg53* cDNA. The tPA-MG53 sequence was cloned behind the chicken beta-actin promoter to drive the expression of the transgene.

Western blot analysis showed elevated levels of MG53 protein in sera derived from the tPA-MG53 mice compared to wild type littermates (Fig. 1a). Specificity of the antibody used to quantify serum levels of MG53 is presented in Supplementary Fig. 1. The enhanced MG53 secretion in the bloodstream of the tPA-MG53 mice was maintained at different ages ranging from 2 months (young), 10–12 months (middle), to 22–24 months (old). On average, the serum level of MG53 in the tPA-MG53 mice was ~120-fold higher than that of the wild-type littermates (Fig. 1b). Quantitative measurement showed that the serum level of MG53 in the tPA-MG53 mice was 5997.1 ± 2071.0 ng/ml (*n* = 9), and the serum level in the wild type mice was 50.4 ± 32.8 ng/ml (*n* = 10). ELISA determination confirmed the elevated MG53 levels in sera derived from the tPA-MG53 mice (Supplementary Fig. 2).

In contrast to the marked elevation of MG53 in the bloodstream, skeletal and cardiac muscles derived from the tPA-MG53 mice showed only a marginal increase over the littermate wild type mice (Fig. 1c). In addition to the native band of a 53 kDa protein, a higher molecular weight protein above the 53 kDa band was observed in western blot with skeletal muscle derived from the tPA-MG53 mice (Fig. 1c). This likely represents the tPA-MG53 protein without cleavage of the tPA secretory peptide which has not undergone processing for protein secretion. We also conducted western blot of MG53 expression in non-muscle tissues, e.g. brain, liver, kidney, and lung, and found measurable accumulation of MG53 in these tissues (Supplementary Fig. 3).

The tPA-MG53 mice lived a healthy life span with no observable pathology in all major vital organs. Three out of five mice survived over 36 months, whereas all wild type control mice died before the age of 30 months. For those tPA-MG53 mice that were sacrificed at 32 months, histological analyses did not reveal obvious pathological changes in any vital organs (Fig. 1d). Additional H/E staining of tissue samples derived from wild type and tPA-MG53 mice at different ages were presented in Supplementary Fig. 4. Thus, sustained elevation of MG53 in the bloodstream did not have detrimental effects on whole body function. In treadmill testing, the tPA-MG53 at the age of 32 months could run at a performance level comparable to young wild type mice (6 months) (See Supplementary Movie 1).

Previously, we demonstrated that intravenous injection of 1 mg/kg rhMG53 could achieve transient elevation of MG53 in the bloodstream to a level of 10–20 µg/ml, which decays with a half-life time of ~1 h in the bloodstream^18^. Such systemic administration of rhMG53 could protect against acute injuries to skeletal muscle, heart, kidney, and lung in animal model studies^13,14,17–19^. The serum level of MG53 in the tPA-MG53 mice (~2–6 µg/ml) is comparable to the level of exogenous rhMG53 achieved via intravenous administration. This suggests that therapeutic approaches with chronic elevation of MG53 in the bloodstream will likely be safe.

### tPA-MG53 mice act normally to glucose and insulin challenges

As a member of the TRIM-family protein, MG53 contains E3-ligase activity which may participate in modulation of metabolic function^20,21^. Song et al.^20^ reported that the MG53 knockout mice displayed a phenotype of lean body mass under normal diet conditions and were resistant to high-fat-diet (HFD) induced body growth with improved glucose handling after HFD treatment. However, this observation has not been recapitulated by other investigators. Specifically, Yi et al.^21^ reported similar body-growth with the wild type and *mg53−/−* mice following HFD treatment (see supplementary Fig. 14 in Yi et al.^21^). In Supplementary Fig. 5, we presented data to show that the *mg53−/−* mice compared to wild type mice showed a trend of increased body weight under normal diet conditions.

**Fig. 2:**
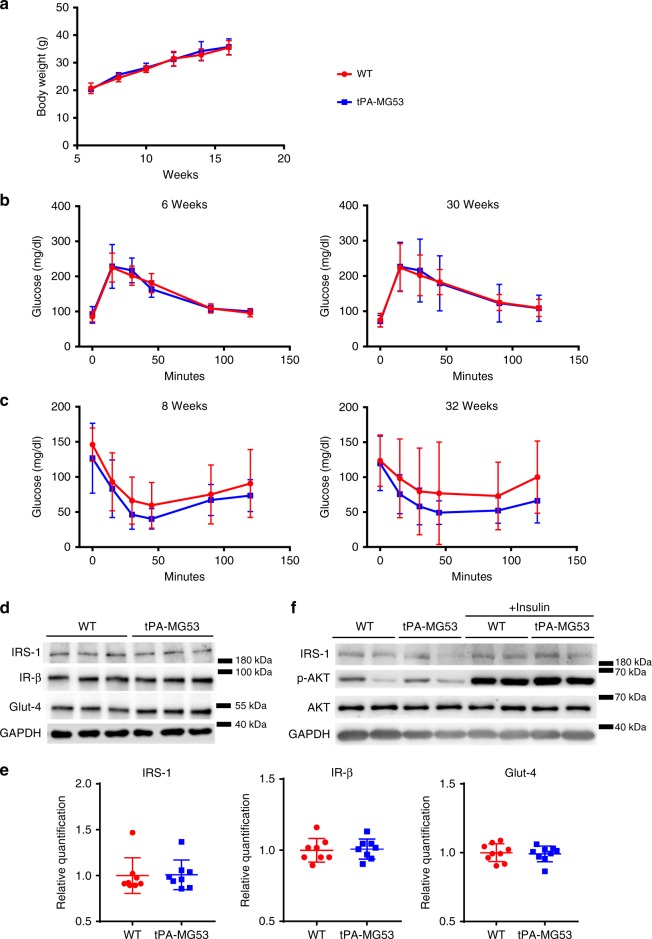
Assessment of insulin signaling and glucose handling in tPA-MG53 and WT mice. **a** tPA-MG53 and WT littermate mice at 6 weeks were treated with HFD and the changes in body weight were followed for 10 weeks (*n* = 5 per group). **b** Glucose tolerance tests were conducted with tPA-MG53 and WT littermates at the age of 6 weeks (left) and 30 weeks (right). **c** Insulin-tolerance tests were conducted with tPA-MG53 and WT littermates at the age of 8 weeks (left) and 32 weeks (right). *n* = 6 for WT, *n* = 5 for tPA-MG53. **d** TA muscle (60 µg total protein per lane) derived tPA-MG53 and WT littermates were probed with antibodies against IRS-1, IR-β, Glut-4. GAPDH serves as loading control. **e** Quantification of protein expressions based on western blot. **f** tPA-MG53 and WT mice were treated with insulin (0.75 U/kg) for 15 min, fresh TA muscles were probed with antibodies against IRS-1, p-Akt, Akt, and GAPDH. Error bar represents the standard deviation

With the tPA-MG53 mice, we did not observe any significant difference in their growth pattern compared to wild type littermates when subjected to HFD treatment (Fig. 2a). We used glucose-tolerance test (GTT) and insulin-tolerance test (ITT) to evaluate if tPA-MG53 mice exhibit any alterations in glucose handling. When mice were challenged with a bolus intraperitoneal injection of glucose (1 g/kg), similar glucose handling was observed between tPA-MG53 and wild type littermates at 6 weeks and 30 weeks of age (Fig. 2b). Moreover, no significant changes in ITT were observed between wild type and tPA-MG53 mice at 8 weeks and 32 weeks of age (Fig. 2c). Data with ITT measurement of mice at 12 weeks age is presented in Supplementary Fig. 6. This data suggests that sustained elevation of MG53 in the bloodstream did not have a significant impact on glucose handling.

We next performed western blot analyses of skeletal muscle tissues derived from the wild-type and tPA-MG53 mice, focusing on key protein components involved in insulin signaling, e.g., IRS-1, insulin receptor β (IR-β), and Glut-4. Glut-4 is the major membrane transporter that facilitates insulin-mediated glucose uptake into skeletal muscle. As shown in Fig. 2d, comparable levels of IRS-1 and IR-β were detected in skeletal muscle derived from wild type and tPA-MG53 mice. Furthermore, there was no measurable changes in Glut-4 protein expression in skeletal muscle derived from the tPA-MG53 mice (Fig. 2e). These measurements were repeated in multiple experiments (*n* = 6–10 mice, 4–16 months of age) (Fig. 2e), and observed in both slow twitch and fast twitch skeletal muscles. Previous studies by Nguyen et al.^31^ show that focal adhesion kinase (FAK) is a potential E3-ligase substrate for MG53. We conducted western blot analysis and found that the protein level of FAK was not altered by MG53 in the tPA-MG53 muscle (Supplementary Fig. 7). In addition, we found ERK expression in wild type and tPA-MG53 muscle also remained similar (Supplementary Fig. 7).

To further test the role of MG53 in insulin signaling, we treated tPA-MG53 mice and their wild type littermates with insulin (0.75 U/kg, intraperitoneal). As shown in Fig. 2f, activation of Akt by insulin was robust and similar in both tPA-MG53 and wild type muscles. These results suggest that overexpression of MG53 does not alter insulin signaling in skeletal muscle.

We also did not observe any changes in PPARα expression level in skeletal muscle derived from the tPA-MG53 mice when compared with the wild type muscle (Supplementary Fig. 7). Overall, these data indicate that mice with sustained elevation of MG53 in the blood stream did not show signs of metabolic dysfunction.

### tPA-MG53 mice show enhanced repair capacity after injury

Previously, we showed that although MG53 is absent from keratinocytes and fibroblasts, remarkable defects in skin architecture were observed in *mg53−/−* mice, and these animals display delayed wound healing and abnormal scarring^32^. MG53 present in circulation may contribute to the maintenance of skin architecture under physiological conditions. Here we used an ear punch model, which has been widely used for mammalian wound repair and regeneration^33^, to assay if increased levels of MG53 in the bloodstream can rejuvenate tissue wound healing capacity.

**Fig. 3:**
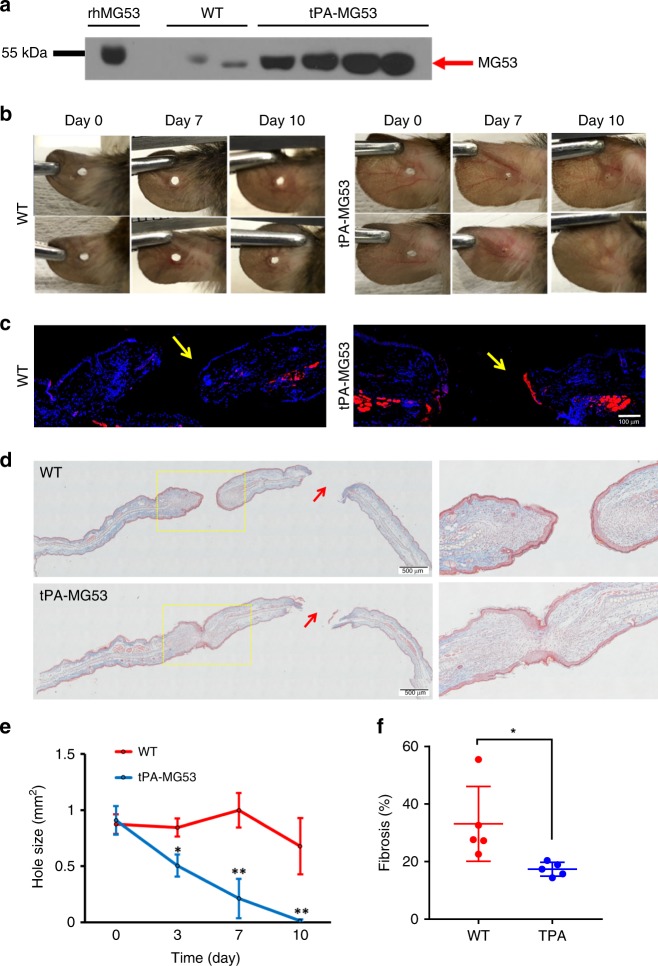
tPA-MG53 mice show increased healing capacity following ear-punch injury. **a** tPA-MG53 mice in a mixed genetic background of 129/Sv and C57BL/6J expressed elevated levels of circulating MG53 compared with WT littermates. 1.3 µl sera were loaded per lane and probed with anti-MG53 antibody. 0.1 ng of rhMG53 dissolved in serum derived from *mg53−/−* mice was used as reference standard. **b** Representative pictures of ear punch injury in WT (left panels) and tPA-MG53 mice (right panels) at different days post-injury. **c** IHC revealed the concentration of MG53 at the leading edge of ear-punch at 2 h after wounding in the tPA-MG53 mice. **d** Masson’s trichrome staining showed remarkable differences in the ear skin architecture between WT and tPA-MG53 mice, with wounds created acutely at 2 h (red arrow) and 14 days (yellow rectangle) following ear punch. **e** WT mice do not heal over the 10-day observation, the tPA-MG53 mice all healed completely at day 10. *n* = 10 per group. **f** Areas positive for trichrome staining were used as index for tissue fibrosis. Quantification revealed significant difference between WT and tPA-MG53 mice at 14 days post ear-punch injury. *p < 0.05; **p < 0.01. Error bar represents the standard deviation and *p* value was generated by *t* test

For this study, a separate tPA-MG53 mouse line with mixed genetic background of 129/Sv and C57BL/6J was used. These mice also have elevated circulating MG53 levels (Fig. 3a). A 1-mm through-and-through ear hole was made and monitored for 14 days. The ear-hole closure was photographed on days 0, 7, and 10. As shown in Fig. 3b, tPA-MG53 mice show significantly enhanced repair capacity after ear-punch injury as compared to their wild-type littermates. The wild type mice did not heal over the 10-day observation whereas the tPA-MG53 mice all healed completely prior to day 10 (Fig. 3b, e, *n* = 10). More interestingly, staining of the ear tissue with antibody against MG53 revealed concentration of MG53 at the healing edge of the ear hole derived from the tPA-MG53 mice (Fig. 3c), indicating that MG53 from circulation was trafficked to the injury site to facilitate wound closure. It is also possible that bleeding associated with the lesion could allow for access of MG53 to the injury site.

Noticeably, at 10 days post injury, all tPA-MG53 mice recovered completely with no observable scarring. These phenomena were reproducibly observed in multiple injuries to the ears of the same tPA-MG53 mice. As shown in Fig. 3d, trichrome staining showed clear differences in the ear skin architecture between wild type and tPA-MG53 mice, with both wounds created acutely at 2 h and 14 days following ear punch. Quantitative analysis demonstrated significant reduction in fibrosis associated with healing of the ear-punch injury in the tPA-MG53 mice when compared with the littermate wild-type mice (Fig. 3f). From these studies, we conclude that increased levels of MG53 in circulation enhances the tissue wound healing capacity.

### Increased performance and regeneration with tPA-MG53 muscle

**Fig. 4:**
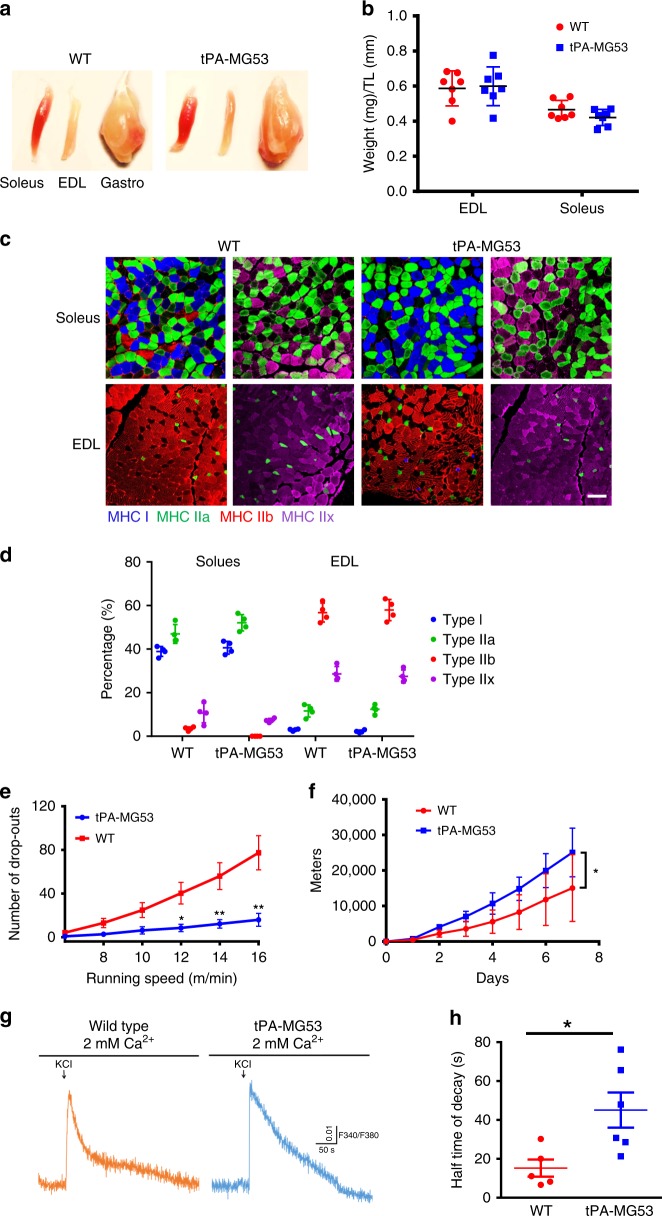
Increased muscle performance of the tPA-MG53 mice under stress conditions. **a** Gross anatomy of soleus, EDL and gastrocnemius muscles derived from tPA-MG53 and WT mice at 3–4-months age. **b** Quantification of the ratio of muscle weight to tibia length (TL) showed no significant differences between WT and tPA-MG53 mice. **c** Cross section IHC staining of soleus and EDL skeletal muscle from WT and tPA-MG53 mice. Green—MHCIIA to stain fast-twitch fiber. Red—MHCIIB another antibody to stain fast twitch fiber. Magenta—MHC I to stain slow twitch fiber. Purple—MHC IIx to stain fast twitch muscle fiber. **d** Fiber typing staining results were quantified. **e** 20 h after the first round of exercise training (10 m/min), mice were again subjected to running at 6, 8, 10, 12, 14, 16 m/min each for 3 min on the treadmill to test the capacity of recovery from muscle injury. The number of drop-outs were quantified. **f** tPA-MG53 and WT mice were subjected to voluntary wheel running for 7 days. *p < 0.05; **p < 0.001. **g** FDB fibers derived from WT (left) and tPA-MG53 mice (right) were loaded with Fura-2 AM; KCl-induced changes in intracellular Ca^2+^ were measured with 2 mM Ca^2+^ present in the extracellular solution. **h** Half-decay times of intracellular Ca^2+^ signaling were significantly longer in tPA-MG53 muscle compared with WT muscle (*p* = 0.0215). Error bar represents the standard deviation and *p* value was generated by *t* test

We conducted experiments to characterize the function of skeletal muscle with the tPA-MG53 mice. Gross anatomy of the soleus, extensor digitorum logus (EDL), and gastrocnemius (Gastro) muscles appears to be comparable between tPA-MG53 and wild-type littermates (Fig. 4a). Quantification of the ratio of muscle weight to tibia length showed no muscle atrophy or hypertrophy in the tPA-MG53 mice (Fig. 4b, *n* = 7). H/E staining of muscle cross section did not reveal detectable pathological changes with the tPA-MG53 muscle. Moreover, cross sectional staining of soleus and EDL muscles with antibodies specific for myosin heavy chain type 1 (MHC1, blue, Fig. 4c), myosin heavy chain type 2a (MHC2a, green, Fig. 4c), myosin heavy chain type 2b (MHC2b, red, Fig. 4c) and myosin heavy chain type IIx (MHC2x, purple, Fig. 4c) did not reveal significant muscle-fiber type switch with the tPA-MG53 mice (Fig. 4d). The amount of MHC2b, which was present in soleus muscle as a minor component in wild-type muscle, was undetectable in the tPA-MG53 soleus (Fig. 4b).

The tPA-MG53 and littermate wild type-mice were subjected to treadmill training to test if elevation of circulating MG53 levels could impact the animals’ running capacity. For this study, mice were first subjected to running at 10 m/min for 6 h. Twenty hours after the first round of exercise training, mice were again subjected to running at various speeds for 3 min on the treadmill to test the capacity of recovery from exercise-induced muscle injury. The number of times the mice fail to run forward and touch the bottom of the electric grid of the treadmill (remaining there for >7 s) was recorded as drop-out. As shown in Fig. 4e, the wild-type mice had significantly more difficulty on the treadmill as they displayed more drop-outs, in particular at higher running speeds, than the tPA-MG53 mice. This observation indicates that exercise-induced muscle injury may be less in the tPA-MG53 mice, and their muscles may also recover faster in the presence of sustained elevation of MG53 in blood circulation.

To evaluate if sustained elevation of MG53 affects the basal performance of mice, the mice were subjected to voluntary wheel running for 7 days. As shown in Fig. 4f, the tPA-MG53 mice showed enhanced running capacity compared to wild type littermate controls (*n* = 5 per group), which did not reflect changes in glycogen content of the muscle fiber since biochemical studies revealed only marginal increase in glycogen content in gastrocnemic muscle derived from tPA-MG53 mice compared with wild-type littermates (see Supplementary Fig. 8). We measured inflammatory cytokines in blood before and after voluntary wheel running, and did not observe any difference in the levels of cytokines between tPA-MG53 and wild-type littermates (Supplementary Fig. 9). Moreover, electron microscopy analysis did not show measurable changes in mitochondria content in both EDL and soleus muscles derived from the tPA-MG53 mice compared with wild-type littermates (see Supplementary Fig. 10).

Previously we reported that MG53 plays a role in modulation of Ca signaling in skeletal muscle^34^. To test if sustained elevation of MG53 impacts Ca signaling in skeletal muscle, we isolated flexor digitorum brevis (FDB) muscle from wild-type and tPA-MG53 littermates. As shown in Supplementary Fig. 11, KCl-induced intracellular Ca release is comparable between wild type and tPA-MG53 muscle fibers in the absence of extracellular Ca. Interestingly, with 2 mM Ca present in the extracellular solution, sustained Ca release was observed in muscle fiber derived from the tPA-MG53 mice (Fig. 4g). The half-time for Ca decay was 45.1 ± 9.0 s (tPA-MG53), and 15.2 ± 4.4 s (wild type, *p* < 0.05) (Fig. 4h), suggesting that increased MG53 expression could enhance extracellular Ca entry in skeletal muscle^34^. This may be a contributing factor for the enhanced muscle performance of the tPA-MG53 mice. Further studies will be needed to ascertain the biological base of this observation.

**Fig. 5:**
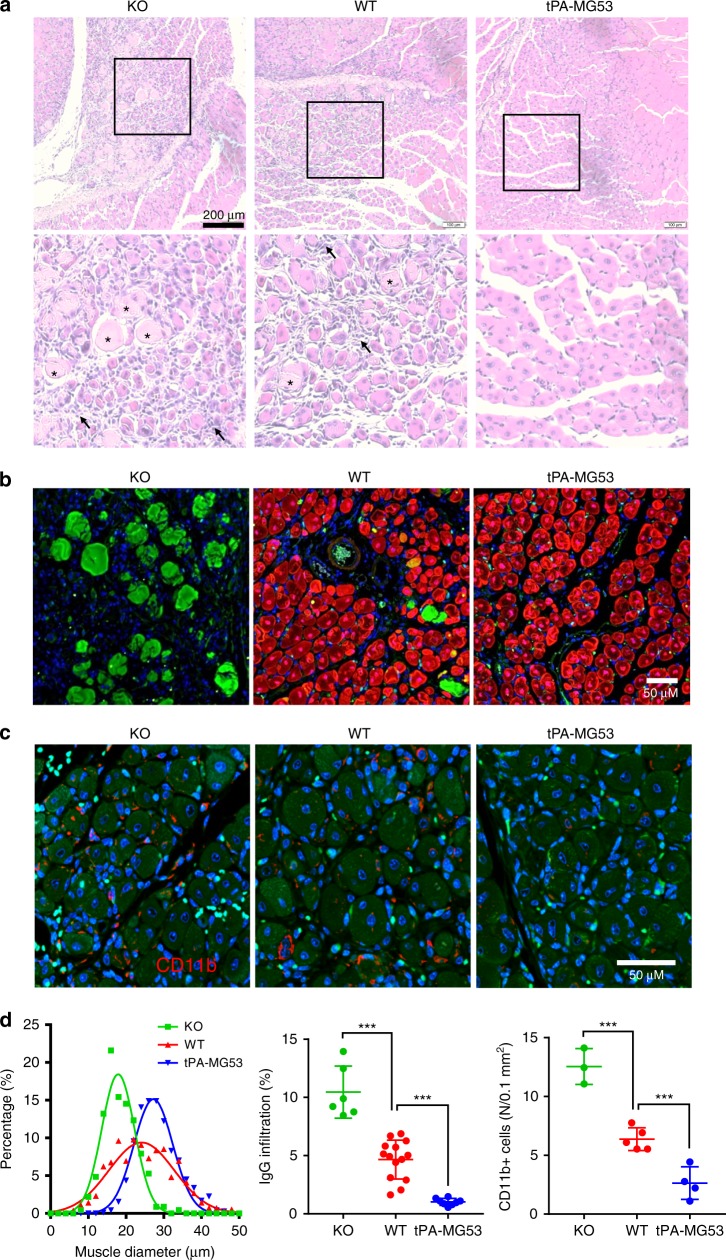
tPA-MG53 skeletal muscle showed enhanced regeneration capacity after cardiotoxin injury. **a** H/E staining of gastrocnemius muscle derived from KO, WT, and tPA-MG53 mice at 7 days post cardiotoxin injury. Enlarged pictures show different regenerative capacity of muscle fibers derived from WT, KO, and tPA-MG53 mice. **b** IHC staining of MG53 (red) and mouse IgG (green). IgG positive staining, mostly in KO muscle, indicates necrotic muscle fibers after cardiotoxin injury. **c** IHC staining with antibody against CD11b to show the different degrees of the presence of immune cells at the muscle injury sites (7 days post cardiotoxin treatment). **d** Quantification of muscle fiber size (left), IgG staining of necrotic fibers (middle) and number of cells positive for CD11b (right). ***p < 0.001, **p < 0.01. Error bar represents the standard deviation and *p* value was generated by *t* test

We next conducted cardiotoxin-induced muscle injury to evaluate the regenerative capacity of the tPA-MG53 muscle^13^. Cardiotoxin (50 µl of 10 µM stock solution) was injected intramuscularly into the hind limb muscle of the wild-type, *mg53−/−*, and tPA-MG53 mice. Seven days after cardiotoxin injury, H/E staining was used to evaluate muscle pathology. As shown in Fig. 5a, there were clear differences between the three muscle samples. The tPA-MG53 muscle showed more homogenous fiber sizes with central nuclei (right panel, Fig. 5a), indicating enhanced muscle regeneration, when compared with those from the wild type (middle panel). The *mg53−/−* muscle fibers (left panel, Fig. 5a) showed more heterogeneous fiber sizes. In addition, reduced accumulation of immune cells were clearly visible within the tPA-MG53 muscle at 7 days post cardiotoxin injury. We used IgG labeling to quantify the extent of muscle necrotic death. As shown in Fig. 5b, more IgG positive staining was observed in *mg53−/−* muscle than in the wild-type and tPA-MG53 muscle, which is consistent with the reduced membrane repair function of muscle fibers with ablation of *mg53*.

To quantify the degree of immune cell infiltration of the injured muscle fibers, we did IHC staining of CD11b. As shown in Fig. 5c, the tPA-MG53 muscle contained significantly less staining of CD11b than the wild type muscle. The *mg53−/−* muscle fiber contained the most CD11b staining. Data from multiple experiments are summarized in Fig. 5d.

**Fig. 6:**
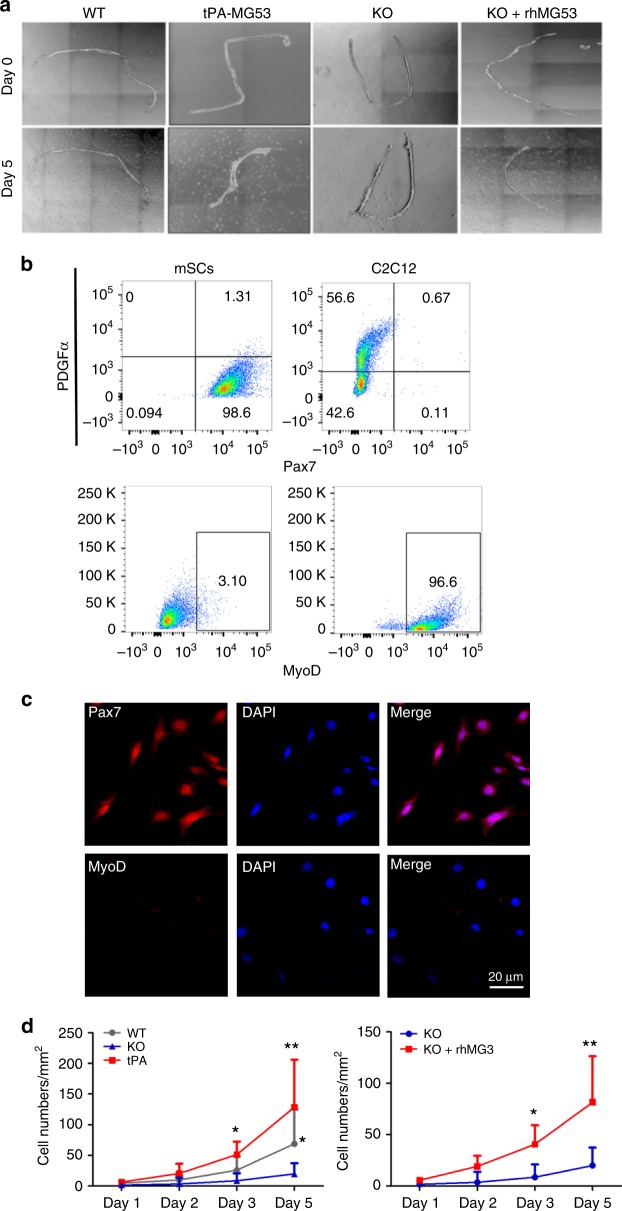
MG53 modulates muscle satellite cell (mSC) proliferation to contribute to muscle regeneration. **a** Outgrowth of mSCs from isolated EDL muscle fibers derived from adult wild-type, tPA-MG53 and KO mice. At day 5 after culture, there were fewer mSCs growing out from KO muscle fibers, as compared to those from wild type and tPA-MG53 muscle fibers. Incubation with exogenous rhMG53 (20 μg/ml) led to increased number of mSCs near the KO myofiber (KO + rhMG53). **b** Flow cytometry analysis with different cell lineage antibodies (Pax7 for mSCs, MyoD for myoblasts, and PDGFα for fibroblasts) to validate the identity of the cultured mSCs. **c** Immunofluorescent staining confirmed the high purity of muscle-fiber derived mSCs, as they are positive for Pax7 and negative for MyoD. **d** Statistical analysis demonstrate that the compromised proliferation of mSCs derived from KO muscle could be restored by incubation with rhMG53 (*n* = 3–6 per condition, *p < 0.05 **p < 0.01 as compared to mg53*−/−* fibers). Error bar represents the standard deviation and *p* value was generated by *t* test

The remarkable regenerative capacity of the tPA-MG53 muscle following cardiotoxin injury suggests the possibility that MG53 may modulate muscle satellite cell (mSC) function to contribute to muscle regeneration. As shown in Fig. 6a, single extensor digitorum longus (EDL) muscle fibers were isolated from adult wild type, tPA-MG53 and *mg53−/−* (KO) mice (4–6 month age), and cultured to allow for outgrowth of mSCs from myofibers. There were fewer mSCs growing out from KO muscle fibers, as compared to those from wild type and tPA-MG53 muscle fibers. More mSCs were found near tPA-MG53 fibers at 5 days after culture. When exogenous rhMG53 (20 μg/ml) was added to the culture medium, an increased number of mSCs were observed near the KO myofiber (Fig. 6a, right). These data provide evidence for a direct effect of MG53 on mSC proliferation. We performed flow cytometry analysis with different cell lineage antibodies (Pax7 for mSCs, MyoD for myoblasts, and PDGFα for fibroblasts) to validate the identity of the cultured mSCs. mSCs were positive for Pax7 and negative for MyoD and PDGFα (Fig. 6b, left), while C2C12 myoblasts were positive for MyoD and negative for Pax7 (Fig. 6b, right) (graphically account for all FACS sequential gating/sorting strategies were summarized in supplementary Fig. 12). Immunofluorescent staining confirmed the high purity of muscle-fiber derived mSCs, as they are positive for Pax7 and negative for MyoD (Fig. 6c). Mean values of the data in Fig. 6a were summarized in Fig. 6d. Clearly, there was a defective mSC proliferation from *mg53−/−* muscle and significantly improved mSC proliferation in tPA-MG53 muscle, compared with mSC derived from WT muscle. More importantly, we found that incubation of *mg53−/−* muscle fibers with rhMG53 protein could restore mSC proliferation. Identification of a role of MG53 in modulation of mSC function provides a mechanistic base for the increased regenerative capacity associated with muscle injury.

### rhMG53 protects against acute and chronic muscle injury

**Fig. 7:**
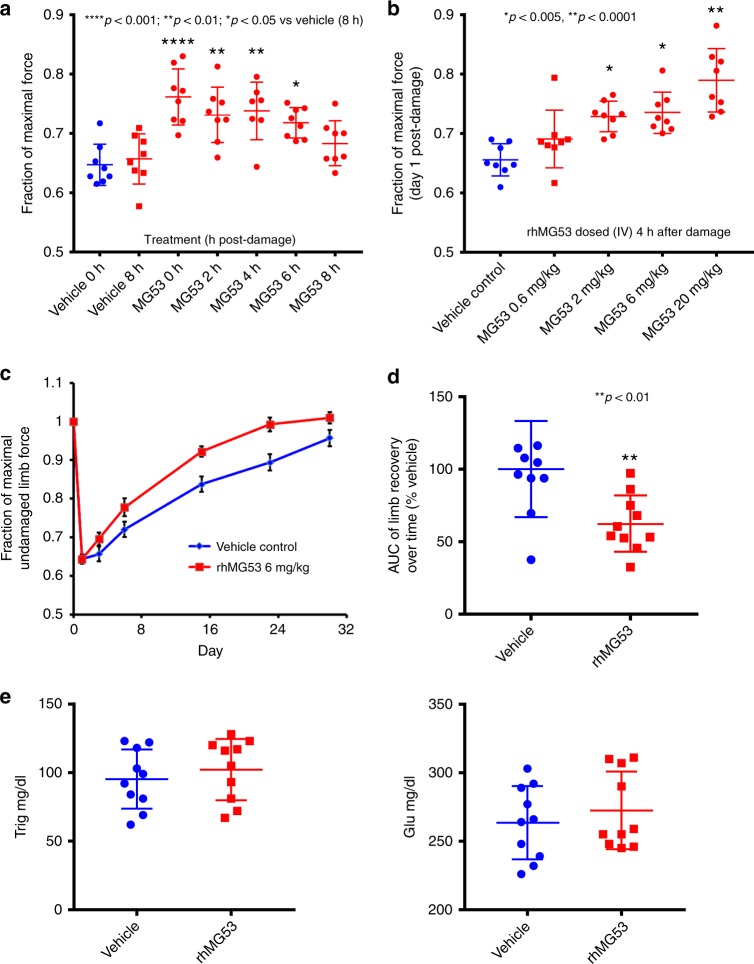
rhMG53 protects against eccentric-contraction-induced acute and chronic muscle injury in mice. **a** Time-dependent effects of rhMG53 in protection against eccentric-contraction-induced acute muscle injury in mice. rhMG53 (2 mg/kg, i.v.) was administered at different times after muscle injury. **b** Dose-dependent effect of rhMG53 in protection against eccentric-contraction-induced acute muscle injury in mice. Various doses of rhMG53 were administered at 4 h post muscle injury. **c** 24 h after eccentric-contraction-induced muscle injury, rhMG53 (6 mg/kg) was administered subcutaneously. Mice were treated on a daily basis for 28 days. **d** Area under the curve (AUC) shows significant beneficial effects of rhMG53 in facilitation of recovery of contractile function after eccentric-contraction-induced muscle injury (*p* < 0.001, compared with saline control). **e** Blood glucose and triglyceride measurement did not show significant changes after 28 doses of 6 mg/kg (s.c., daily) in mice. Error bar represents the standard deviation and *p* value was generated by *t* test

To gain further insight into the direct action of circulating MG53 in protection against chronic muscle injury, we conducted an extensive series of studies using eccentric-contraction to induce muscle injury in mice (Fig. 7). The hindlimb muscle (plantar flexors) of C57BL/6N mice (male, 12–14 weeks of age) were stimulated to produce tetanic contractions (via sciatic nerve stimulation, 60 repetitions, 10 s apart) while being forcibly lengthened in vivo. Following the eccentric contraction-induced muscle injury, mice were administered with rhMG53 (2 mg/kg) via tail vein injection at different times after muscle injury (Fig. 7a). We found that intravenous administration of rhMG53 protects acute muscle injury in a time-dependent manner. Even at 6 h post muscle injury, rhMG53 is still effective (*p* < 0.05). Based on the finding that significant improvement in muscle contractility was observed with rhMG53 at 4 h post-muscle injury (Fig. 7a), subsequent experiments were performed with treatment of mice at 4 h post-muscle injury with varying doses of rhMG53 (0, 0.6, 2, 6, and 20 mg/kg, intravenously). As shown in Fig. 7b, rhMG53 protein protected against eccentric contraction-induced muscle injury in a dose-dependent manner.

We next conducted a longitudinal evaluation of rhMG53 to potentially protect against chronic muscle injury in mice (Fig. 7c). Longitudinal measurement of muscle contractility was performed at day 0 (baseline), and day 1, 3, 6, 15, 23, and 30 post-injury in mice treated with vehicle control (blue color) or rhMG53 protein (red color). Mice were treated with rhMG53 subcutaneously (6 mg/kg) on a daily basis for 4 weeks. This first dose of rhMG53 was administered at 24 h after eccentric contraction-induced muscle injury. If rhMG53’s sole function was to protect against sarcolemma injury, one would not expect to see any beneficial effects of rhMG53 at 24 h post-musfcle injury when all injured muscle fibers would have died. However, we were excited to see that repetitive dosing of rhMG53 at 24 h post muscle injury still has therapeutic benefits (Fig. 7c). Area under the curve (AUC) shows significant beneficial effects of rhMG53 in facilitation of recovery of contractile function after eccentric-contraction-induced muscle injury during the 28-day observation period (Fig. 7d, *p* < 0.001, compared with saline control).

This unexpected finding provides evidence for a direct role of MG53 in protection against chronic muscle injury which is likely linked to facilitation of mSC function. Moreover, we found repetitive dosing of rhMG53 is safe and did not alter glucose handling (Fig. 7e), an observation that was consistent with the healthy lifespan of the tPA-MG53 mice which contained >100-fold elevation of MG53 in their bloodstream.

### *db/db*-tPA-MG53 mice do not alter insulin/glucose handling

**Fig. 8:**
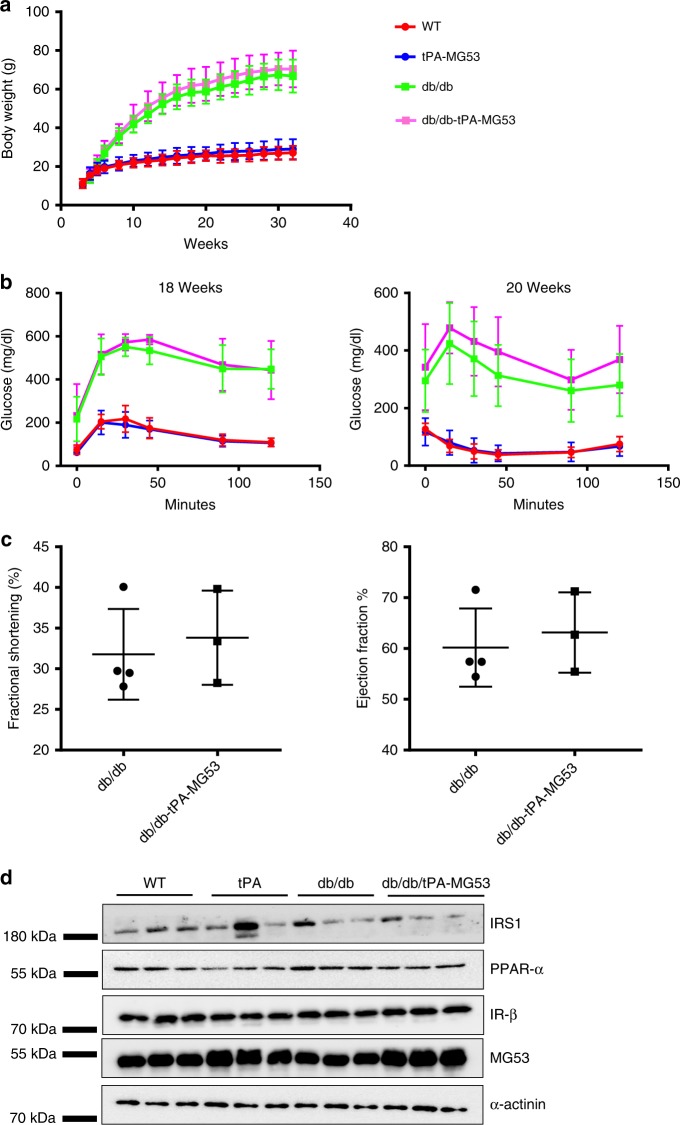
Cross of tPA-MG53 mice with db/db mice did not alter the diabetic phenotype. **a** Growth patterns of WT and tPA-MG53 littermates; and *db/db* and *db/db*-tPA-MG53 littermates were followed during the period of 3–32 weeks, under normal diet conditions. **b** GTT tests were conducted with the four groups of mice at 18 weeks of age (left). ITT tests were conducted at 20 weeks of age. No significant differences were observed among the WT and tPA-MG53, and *db/db* and *db/db*-tPA-MG53 groups, respectively. *n* = 6 for WT, *n* = 5 for tPA-MG53, *n* = 6 for *db/db*, and *n* = 5 for *db/db-*tPA-MG53. **c** Echocardiogram shows similar ejection fraction and fractional shortening between *db/db*-tPA-MG53 and *db/db* littermates. **d** Western blot of soleus muscle derived from the four groups of mice. Expression levels of IRS-1, IR-β, and PPARα were probed with the respective antibodies. α-actinin serves as loading control. Error bar represents the standard deviation

The tPA-MG53 mice were crossed with *db/db* mice in order to evaluate if sustained elevation of MG53 in circulation impacts metabolic functions (Supplementary Fig. 13). As shown in Fig. 8a, the *db/db*-tPA-MG53 mice displayed similar growth pattern as *db/db* mice under normal diet condition. Moreover, there were no differences in growth patterns between tPA-MG53 and wild type littermates. We conducted GTT and ITT tests with the four groups of mice: wild type, tPA-MG53, *db/db*, and *db/db*-tPA-MG53, at the age of 18–20 weeks. As shown in Fig. 8b, the glucose tolerance of *db/db* and *db/db*-tPA-MG53 mice were indistinguishable, as were their insulin tolerance. Consistent with the results shown in Fig. 2b, c, we detected no changes in GTT and ITT between wild type and tPA-MG53 at 18–20 weeks of age.

We used echocardiography to determine if sustained elevation of MG53 in the *db/db* mice could alter their cardiac function. As shown in Fig. 8c, the ejection fraction and fractional shortening of the heart were similar in *db/db*-tPA-MG53 and *db/db* mice at 8–10 weeks of age. Thus, increased MG53 levels in circulation did not have any deleterious effects on heart function.

These findings are surprising, as they contradict the previous reports by Song et al.^20^ and Liu et al.^30^. If their hypothesis is correct, we would expect to see drastic exaggeration of the metabolic defects in the *db/db*-tPA-MG53 mice due to the augmented MG53 action on IRS-1 and PPARα mediated insulin signaling and lipogenic function. We thus conducted western blot analyses with soleus muscle derived from *db/db* and *db/db*-tPA-MG53 mice. As shown in Fig. 8d, the protein levels for IRS-1, PPARα, and IR-β did not show clear differences between the two groups.

Liu et al.^30^ showed that mice with cardiac-specific overexpression of MG53 led to nuclear translocation of MG53, which can activate PPARα transcription by binding to the PPARα promoter region. Activation of the PPARα led to lipogenesis in cardiomyocytes, resulting in cardiomyopathy. Interestingly, although we did observe nuclear accumulation of MG53 in tPA-MG53 muscle (see Supplementary Fig. 14), we failed to observe detectable changes in the PPARα in our mouse lines.

Overall, our findings present the evidence that elevated MG53 levels in circulation is safe to metabolic and cardiac function.

## Discussion

In the present study, we used a transgenic mouse line (tPA-MG53) with sustained elevation of MG53 protein in blood circulation to evaluate the physiological function of MG53 in tissue injury-repair and regeneration. The tPA-MG53 mice maintain high levels of circulating MG53 throughout their lifespan (up to 36 months) with no observable side effects. In addition to the prolonged lifespan, tPA-MG53 mice display a remarkable regenerative capacity in response to injurious assaults to multiple organs. When challenged with treadmill running and cardiotoxin injury, the tPA-MG53 mice displayed improved exercise performance and enhanced healing ability in response to muscle injury. More excitingly, while the skin tissues do not contain endogenous MG53 protein, ear punch injury to the tPA-MG53 mice can heal remarkably faster than wild type mice. Biochemical and imaging analyses reveal that MG53 can travel in the bloodstream to concentrate on the healing edge of the ear wound sites, thus contributing to the improved regenerative capacity following tissue injury. Together, our data supports the safety of elevated levels of MG53 circulating in our bodies under physiological conditions, and the beneficial effects of MG53 in the bloodstream on tissue injury-repair and regeneration. Thus, a therapeutic approach with intravenous administration of rhMG53 may represent a safe and effective means to treat multiple diseases caused by tissue injuries.

Since our identification of MG53 as a key component of cell membrane repair in 2009, many research groups have joined in this important research area. We know that genetic ablation of MG53 leads to defective cell membrane repair, which can cause progressive skeletal myopathy and decreased regenerative capacity of cardiomyocytes. To evaluate the in vivo functions of MG53, multiple strategies have been employed. Researchers have used virus based delivery systems to overexpress MG53 in both skeletal muscle^35^ and hearts^12^, and found that overexpression of MG53 can protect against injuries to muscle and hearts and ameliorate muscular dystrophy and cardiomyopathy in animal models.

In addition to virus mediated gene delivery, a transgenic approach has also been used by multiple groups to test the physiological function of MG53. Prior to our study with the tPA-MG53 mice, three different lines of MG53 transgenic mice were reported in the literature^21,30,36^. Xiao and colleagues used both a chicken β-actin promotor^21^ and α-myosin heavy chain promotor^30^ to drive MG53 expression in skeletal muscle and the heart respectively. They reported that overexpression of MG53 caused metabolic disorders in mice through down regulation of IRS-1, an E3 ubiquitin substrate of MG53^21^. With the cardiac-specific MG53 transgenic mice, Liu et al. showed that, in addition to IRS-1 downregulation, MG53 can be found in the nucleus of cardiomyocytes to modulate transcriptional activation of PPARα signaling^30^, and the elevated PPARα activity could contribute to the hypertrophy and cardiomyopathy observed in their mouse cohort. Interestingly, separate studies by Ham and Mahoney reported that cardiac-specific MG53 transgenic mice first showed hypotrophy of the heart at a young age, and only with aging did the transgenic animals begin to develop cardiac hypertrophy^36^. Contrary to the reduced IRS-1 level in heart tissues derived from the MG53 transgenic mice reported by Liu et al., Ham and Mahoney showed elevated IRS-1 expression with their transgenic mice at similar ages^36^.

Our present study took a different approach to assess the function of MG53 by overexpressing secretory MG53 from striated muscles. Our tPA-MG53 mice contain sustained elevations of MG53 in blood circulation at a level that is close to the therapeutic dose of rhMG53 achieved via intravenous infusion in dogs and pigs^18,19^. We did not observe any changes in IRS-1 and IR-β in muscle tissues derived from tPA-MG53 mice. We also failed to detect activation of PPAR-α signaling in both skeletal muscle and heart with our tPA-MG53 mice. The difference between our observations and those of Song et al. and Liu et al. may lie in the distribution of MG53. It is possible that overexpression of MG53 inside the muscle and heart tissues may have deleterious effects as reported by Song et al. and Liu et al. Our finding suggests that increased levels of MG53 in circulation is beneficial to tissue protection, not harmful to their metabolic function.

We present evidence that sustained elevation of MG53 in circulation did not affect insulin signaling and glucose handling in mice. Moreover, the metabolic and cardiac function of *db/db* mice were not altered when they were crossed with the tPA-MG53 mice. These results challenge the suggestion that MG53 functions as a double-edged sword in tissue regeneration and metabolic modulation^37^. Although some potentially detrimental effects from MG53 can take place through intracellular actions on IRS-1 and PPARα signaling pathways, administration of rhMG53 protein through the bloodstream can largely minimize this intracellular action. Due to the relatively short half-life of MG53 protein in blood circulation^13,18^, it is unlikely the administration of MG53 can impact the body’s metabolic function, especially when using the recombinant MG53 protein to treat acute tissue injuries and dermal wounds.

While our manuscript was under review, a paper by Wu et al. was published in Circulation, concluding an immuno-approach using MG53 antibody to reduce its serum level to treat diabetes^38^. They reported that serum derived from the *db/db* mice show elevation of MG53 could be a causative factor for diabetes; and intravenous administration of antibody against MG53 could reduce blood glucose levels in the *db/db* mice. However, the biochemical and biophysical studies used by the authors were not adequate to support their conclusion regarding the physiological role of MG53 and its functional interaction with the insulin receptor for control of glucose metabolism (see letter to the editor by Zhu et al.^39^). While their proposed immuno-therapeutic approach is intriguing, the data presented by the authors contained large standard errors in evaluation of blood glucose levels with the *db/db* mice. Moreover, the authors did not provide any safety data with repetitive dosing of the antibody on vital organ function. Establishing the safety and efficacy of the antibody against MG53 will be of paramount importance if such an immuno-approach were to be of any benefit to diabetic patients.

One main finding of our present study is that sustained elevation of MG53 in circulation leads to increased regenerative capacity of skeletal muscle associated with cardiotoxin and exercise-induced muscle injury. To arrive at a mechanistic understanding of the physiological role of MG53 in modulating muscle regeneration, we isolated mSCs from *mg53−/−*, wild-type, and tPA-MG53 mice, and found defective mSC proliferation from *mg53−/−* muscle and significantly improved mSC proliferation in tPA-MG53 muscle, compared with mSC derived from wild-type muscle. More importantly, we found that incubation of *mg53−/−* muscle fibers with rhMG53 protein could restore mSC proliferation. Identification of a role of MG53 in modulation of mSC function provides a mechanistic base for the increased regenerative capacity associated with muscle injury.

To gain insight into the direct action of circulating MG53 in protection against chronic muscle injury, we conducted experiments using eccentric-contraction to induce muscle injury in mice. We found that intravenous administration of rhMG53 protects acute muscle injury in a time and dose-dependent manner. Even at 6 h post muscle injury, rhMG53 is still effective. These findings are consistent with a role of MG53 in muscle-injury repair; however, we were surprised to see that repetitive dosing of rhMG53 at 24 h post muscle injury still has therapeutic benefits. This unexpected finding provides evidence for a direct role of MG53 in protection against chronic muscle injury which is likely linked to facilitation of mSC function. Moreover, we found repetitive dosing of rhMG53 is safe and did not alter glucose handling, an observation that was consistent with the healthy lifespan of the tPA-MG53 mice which contained >100-fold elevation of MG53 in their bloodstream.

Overall, data presented in this study advance our understanding for MG53 in preservation of the regenerative capacity of skeletal muscle, which is beyond our current knowledge of MG53 as a sole muscle sarcolemma injury-repair molecule. We show that MG53 contributes to the proliferation of mSCs and rhMG53 protein had a sustained impact on chronic muscle injury when administered systemically. These findings shall provide a base for the potential use of rhMG53 to treat chronic muscle diseases associated with the loss of muscle integrity and muscle satellite cell function.

## Methods

### Experimental animals

Animal handling and surgical procedures were performed according to protocols approved by the Institutional Animal Care and Use Committee (IACUC) of The Ohio State University and were compliant with guidelines of the American Association for the Accreditation of Laboratory Animal Care. MG53 knockout mice (*mg53−/−*) and their wild type control mice were bred and maintained as previously described. For generation of the tPA-MG53 mice, human MG53 cDNA was conjugated with a secretory signaling peptide (tPA) at the 5’ end. tPA-MG53 expression construct was generated by cloning tPA-MG53 into an expression vector containing chicken β-actin promoter. Then the plasmid was sent to the Transgenic/Knock-out Mouse (TG/KO) Facility at Cancer Institute of New Jersey (CINJ) for transgenic mouse generation. This transgenic mouse line was generated in mixed genetic background of 129/Sv and C57BL/6 J and was used for the ear punch experiment (Fig. 3). Because it was generated earlier than the other tPA-MG53 mouse line, this mouse line was also used for the aging experiment.

A separate tPA-MG53 mouse line was generated from C57BL/6 J genetic background by Cyagen Biosciences. Santa Clara, CA, USA. This mouse line was used for all experiments except for the ear punch and aging studies. Both tPA-MG53 mouse lines express high levels of circulating MG53 and developed normally.

### Serum MG53 quantification

Two different methods were used to quantify serum MG53 levels in mice. For western blot, different amounts of rhMG53 (0.03, 0.05, 0.1, and 0.3 ng) were mixed with 1 μl of serum from *mg53−/−* mice to serve as a standard. Serum samples from wild-type mouse was loaded with volumes of 1 and 2 μl and probed with a monoclonal antibody against MG53. The density of the western blot was plotted against the rhMG53 standard concentrations, and regression analysis used to calculate the concentration of MG53 in serum derived from the wild-type mice. Similarly, to quantify MG53 in tPA-MG53 mice sera, higher amounts of rhMG53 (1, 5, and 10 ng) were mixed with 1 μl of *mg53−/−* serum to serve as standard. Each serum sample from tPA-MG53 mouse was loaded with volumes of 0.1, 1, and 2 μl. The density of the western blot was plotted against the rhMG53 protein standard concentration and regression analysis used to calculate the concentration of MG53 in tPA-MG53 serum.

MG53 levels in serum were also quantified with a sandwich ELISA assay (Supplementary Fig. 1). Serum samples derived from tPA-MG53 mice were diluted by 50, 200, and 1000-fold in BSA blocking buffer and loaded to an ELISA plate. To generate a standard curve, different amounts of rhMG53 protein spiked into the serum derived from *mg53−/−* mice, and then further diluted with BSA blocking buffer for 50, 200, and 1000-fold and loaded to the same ELISA plate for quantification. The reading of each standard protein was then plotted against its concentration, and regression analysis used to calculate the concentration of MG53 in tPA-MG53 serum.

### Glucose tolerance test

Mice were singly housed and fasted for 16 h prior to tests. The tail tip of the mouse was removed by a sterile scalpel blade. Blood from the tail tip was collected and basal blood glucose was measured by an OneTouch Ultra Glucometer (Lifescan, Milpitas, CA). Following fasting, mice were intraperitoneally injected with a bolus of glucose (1 g/kg, 10% D-glucose, freshly prepared in sterile 0.9% NaCl solution). Blood glucose from mouse tail vein was assessed at 15, 30, 45, 90, and 120 min after glucose administration.

### Insulin tolerance test

Mice were singly housed and fasted for 6 h prior to test. The tail tip of the mouse was removed by a sterile scalpel blade. Blood from the tail tip was collected and basal blood glucose was measured by an OneTouch Ultra Glucometer (Lifescan, Milpitas, CA). Following fasting, mice were intraperitoneally (i.p.) injected with a bolus of insulin (0.75 U/kg) (Sigma, insulin was freshly prepared in sterile 0.9% NaCl solution). Blood glucose was assessed at 10, 25, 45, 90, and 120 min after insulin administration.

### Ear punch injury

A 1-mm ear hole was made in the center of the ear of mice using a metal ear puncher (Fisher Scientific, Cat. No 50822358). The ear holes were photographed and measured at indicated time points using a caliper. At 14 days after ear punch, the mice were sacrificed and ears were dissected for histochemical analysis. The paraffin-embedded ear samples were cut for trichrome staining to quantify fibrosis of regenerated tissues. For acute injury, 2 h after ear punch, the ear samples were collected for immunohistochemical analysis of MG53 at the leading edge of ear wound.

### Treadmill and voluntary-wheel running experiments

tPA-MG53 and wild-type littermates were initially trained (5 m/min running for 5 min each time, running for three times each day for three days) on a small animal treadmill (Columbus Instruments). Then the mice were subjected to treadmill running at 10 m/min for 6 h. Twenty hours after the initial exercise training, mice were subjected to running at 6, 8, 10, 12, 14, and 16 m/min each for 3 min on the treadmill to test the capacity of recovery from muscle injury. The number of times the mice fail to run forward and touch the bottom of the electric grid of the treadmill and remain there for over 7 s was recorded as drop-out. Drop-outs of each mouse at each different speed were recorded.

In separate studies, tPA-MG53 and wild type littermates were individually kept in cages equipped with voluntary free-spinning running wheels (Columbus Instruments, Columbus, OH) for one week. The voluntary running activity were recorded by wheel rotations at 2 h intervals using Windows software (Columbus Instruments, Columbus, OH).

### Ca^2+^ measurement in isolated skeletal muscle fibers

FDB muscle fibers were isolated from wild-type and tPA-MG53 mice following the protocol of Zhu et al.^40^. They were loaded with 10 μM Fura-2 AM. The ratio of Fura-2 fluorescence at excitation wavelength of 340 and 380 nm was measured using a PTI spectrofluorometer (Photon Technology International) to assess the changes in intracellular [Ca^2+^]_i_ following stimulation with KCl. Zero Ca^2+^ or 2 mM Ca^2+^ Tyrode’s solution was perfused onto the fiber before adding 110 mM KCl to induce Ca^2+^ store release.

### Histopathology and immunofluorescent staining

All histopathological and immunofluorescent assays were conducted in a double-blinded manner. Paraffin-embedded tissue sections of 4 µm thickness were used for hematoxylin and eosin (H&E) staining. Tissue samples were fixed in 4% paraformaldehyde (PFA) overnight at 4 °C. After fixation, samples were washed three times for 5 min with 70% ethanol. Washed samples were processed and embedded in paraffin. 4-μm-thick paraffin sections were cut as slides for pathological staining like H&E and Masson trichrome and immunofluorescent staining. Immunofluorescent staining was performed as follows: slides were deparaffinized and rehydrated by incubating successively in xylene, 100% ethanol, 95, 75, 50% ethanol and PBS. Antigen retrieval was achieved by heating in the pressure cooker with Tris-EDTA buffer for 13 mins. Primary antibodies were applied and incubated at 4 °C overnight. Goat anti-rabbit/mouse secondary antibody Alexa-546/Alexa-647 were applied and incubated at room temperature for 1 h. The antibodies used for immunofluorescent staining are: anti-MHC I antibody (Developmental Studies Hybridoma Bank (DSHB), Iowa, BA-F8, 1:50), anti-MHC IIa antibody (DSHB, SC-71, 1:500), anti-MHC IIb antibody (DSHB, BF-F3, 1:100), anti-MHC IIx antibody (DSHB, 6H1, 1:50), anti-CD11b antibody (Abcam, ab133357, 1:200), and anti-MG53 antibody (homemade monoclonal antibody). DAPI was used to stain the nucleus of the tissue. All images were captured by Zeiss LSM 780 confocal microscope and analyzed by ImageJ.

### Immunoblotting

Crude extracts from dissected muscle or heart of experimental animals were washed twice with ice-cold PBS and lysed in RIPA buffer (10 mM Tris-HCl, pH 7.2, 150 mM NaCl, 1% NP-40, 0.5% SDS, and 0.5% deoxycolate), supplemented with a cocktail of protease inhibitors (Sigma) and phosphatase inhibitors (Thermo Scientific). Heart, muscle lysates or serum samples were separated by 10% SDS-PAGE and transferred onto polyvinylidene fluoride membranes (PVDF) (Millipore). The blots were washed with Tris-buffered saline Tween-20 (TBST), blocked with 5% milk in TBST for 1 h, and incubated with custom-made monoclonal anti-MG53 antibody (1:2000)^18,30^ or commercial IRS-1 antibody (Invitrogen, Cat. No. 700662, 1:1000), anti-PPARα antibody (NovusBio, Cat. No. NB600-636, 1:1000), anti-IR-β antibody (Cell Signaling, Cat. No. 3025, 1:1000), and anti-Glut-4 (Cell Signaling, Cat. No. 2213, 1:1000) antibody. Immunoblots were visualized with an ECL plus kit (Pierce).

We routinely used multiple samples derived from the samples in a given western blot. To illustrate the spread of the band intensity with the wild type samples, we normalized the band intensity to one wild type sample (which is often the one with middle intensity), and plotted the relative intensity of other samples (including wild type and tPA-MG53 muscles) in the scatter plot.

### Cardiotoxin induced skeletal muscle injury

At 4–6 months of age, *mg53−/−*, wild type, and tPA-MG53 mice were treated with 50 µl of 10 μM cardiotoxin VII4 (CTX, Sigma-Aldrich) via intramuscular injection. After 7 days, mice were euthanized and skeletal muscle were collected for histological analyses. For all histological analyses, the listed tissues were surgically dissected, fixed in formalin (Electron Microscopy Services) overnight at 4 °C followed by histochemical procedure described above. Analyses were performed double-blinded.

### Eccentric contraction-induced muscle injury in mice

Male C57BL/6N mice at 12–14 weeks of age were individually housed in a 12:12 h (dark: light) cycle, and acclimated to the GSK vivarium for 1 week prior to initiation of muscle injury and intervention. Animals were randomized to treatment groups based on baseline body weight. Mice were anesthetized using isoflurane. Hindlimb muscles were stimulated to produce tetanic contractions (via sciatic nerve; 60 repetitions; 10 s apart) while being forcibly lengthened in vivo. The protocol was approved by the IACUC at GSK. Following eccentric contraction-induced muscle injury, mice were divided into groups of 10 each according to the following experimental designs: a) tail vein administration of rhMG53 (2 mg/kg) at different times post injury; b) tail vein administration at 4 hours post muscle injury with different doses of rhMG53 (0, 0.6, 2, 6 and 20 mg/kg); and c) subcutaneous administration of rhMG53 (6 mg/kg) on a daily basis, with the first dose applied at 24 h post muscle injury. Following the longitudinal study with repetitive dosing of rhMG53, mice were sacrificed at 28 days post injury, and serum glucose and triglycerides were quantified. All measurements were conducted in a double-blinded manner.

### Culture of muscle satellite cells

Extensor digitorum longus (EDL) muscle from *mg53−/−*, tPA-MG53 and their wild type littermates were dissected and digested with 0.2% collagenase at 35 °C for 45 min in a shaking water bath. Single muscle EDL muscle fibers were picked with a heat polished Pasteur pipette and placed at the center of the individual wells of a 24-well matrigel coated plate. The culture media (DMEM plus 20% FBS) was changed every 3 days to allow outgrowth of muscle satellite cells at 37 °C (5% CO_2_). The identity of the cultured satellite cells were confirmed by Pax 7 antibody (Iowa Hybridoma Bank) staining by flow cytometry and immunofluorescent staining. Antibodies against MyoD (myoblast marker) and PDGFα (fibroblast marker) were used to show the purity of isolated satellite cells.

### Echocardiography

Echocardiography was performed in mice anesthetized with 2% isofluorane, using a Vevo2100 echocardiographic system with a 30-MHz transducer (Visualsonics, Inc., Toronto, Ontario, Canada). The heart was first viewed using the two-dimensional mode in the parasternal long-axis and then short-axis views. The short-axis views were used to position the M-mode cursor perpendicular to the ventricular septum and LV posterior wall. LV internal end-diastolic diameters (LVEDD) were measured at the apparent maximal LV diastolic dimensions, and LV internal end-systolic diameters (LVESD) were measured at the most anterior systolic excursions of the posterior wall. LV volumes and ejection fractions (EF) were calculated by the Teichholz method. For volumes, LVEDV = 7(LVEDD)3/(2.4 + LVEDD), and LVESV = 7(LVESD)3/(2.4 + LVESD), where LVEDV and LVESV are the LV end diastolic and systolic volumes respectively. For EF, EF (%) = (LVEDV − LVESV)/LVEDV × 100. LV fractional shortening (FS) was calculated by FS (%) = (LVEDD − LVESD)/LVEDD × 100.

### Statistical analysis

The data are represented as mean ± standard deviation. Comparisons were made by Student’s *t*-test when comparing two experimental groups and by ANOVA for repeated measures. The standard deviation of the mean is indicated by error bars for each group of data. A value of *p* < 0.05 was considered significant. All of these data were analyzed using Prism 5 software.

### Reporting summary

Further information on research design is available in the Nature Research Reporting Summary linked to this article.

## Supplementary information

Supplementary Information

Peer Review

Description of Additional Supplementary Files

Supplementary Movie 1

Reporting Summary

Source Data

## Data Availability

The authors declare that the data of this study are available within the article and its supplementary information files. The source data underlying Figs. 1b, 2a–c, e, 3e–f, 4b, d–h, 5d, 6d, 7a–e, and 8a–c and Supplementary Figs. 2, 5, 6, 7b, 8, 9, 11 are provided as a Source Data file.

## References

[1] Barbee KA (2005). Mechanical cell injury. Ann. N. Y. Acad. Sci..

[2] Cooper ST, Head SI (2015). Membrane injury and repair in the muscular dystrophies. Neuroscientist.

[3] Cooper ST, McNeil PL (2015). Membrane repair: mechanisms and pathophysiology. Physiol. Rev..

[4] Karstoft K, Pedersen BK (2016). Skeletal muscle as a gene regulatory endocrine organ. Curr. Opin. Clin. Nutr. Metab. Care..

[5] Schnyder, S. & Handschin, C. Skeletal muscle as an endocrine organ: PGC-1alpha, myokines and exercise. *Bone* **80**, 115–125 (2015). 10.1016/j.bone.2015.02.008 PMC4657151 26453501

[6] Iizuka K, Machida T, Hirafuji M (2014). Skeletal muscle is an endocrine organ. J. Pharmacol. Sci..

[7] Pratesi A, Tarantini F, Di Bari M (2013). Skeletal muscle: an endocrine organ. Clinical Cases Mineral Bone Metab..

[8] Febbraio MA, Pedersen BK (2005). Contraction-induced myokine production and release: is skeletal muscle an endocrine organ?. Exerc. Sport Sci. Rev..

[9] Cai C (2009). Membrane repair defects in muscular dystrophy are linked to altered interaction between MG53, caveolin-3, and dysferlin. J. Biol. Chem..

[10] Cai C (2009). MG53 regulates membrane budding and exocytosis in muscle cells. J. Biol. Chem..

[11] Tan T, Ko YG, Ma J (2016). Dual function of MG53 in membrane repair and insulin signaling. BMB Rep..

[12] Cao CM (2010). MG53 constitutes a primary determinant of cardiac ischemic preconditioning. Circulation.

[13] Weisleder N (2012). Recombinant MG53 protein modulates therapeutic cell membrane repair in treatment of muscular dystrophy. Sci. Transl. Med..

[14] Zhu H (2015). Amelioration of ischemia-reperfusion-induced muscle injury by the recombinant human MG53 protein. Muscle Nerve.

[15] Yao, Y. et al. MG53 permeates through blood-brain barrier to protect ischemic brain injury. *Oncotarget*, 10.18632/oncotarget.7965 (2016). 10.18632/oncotarget.7965 PMC5008374 26967557

[16] Ma H (2015). Effect of metabolic syndrome on mitsugumin 53 expression and function. PLoS ONE..

[17] Jia Y (2014). Treatment of acute lung injury by targeting MG53-mediated cell membrane repair. Nat. Commun..

[18] Duann P (2015). MG53-mediated cell membrane repair protects against acute kidney injury. Sci. Transl. Med..

[19] Liu J (2015). Cardioprotection of recombinant human MG53 protein in a porcine model of ischemia and reperfusion injury. J. Mol. Cell. Cardiol..

[20] Song R (2013). Central role of E3 ubiquitin ligase MG53 in insulin resistance and metabolic disorders. Nature.

[21] Yi JS (2013). MG53-induced IRS-1 ubiquitination negatively regulates skeletal myogenesis and insulin signalling. Nat. Commun..

[22] Ma LL (2013). Hypercholesterolemia blocked sevoflurane-induced cardioprotection against ischemia-reperfusion injury by alteration of the MG53/RISK/GSK3beta signaling. Int. J. Cardiol..

[23] Yuan H (2013). Proteomic analysis of skeletal muscle in insulin-resistant mice: response to 6-week aerobic exercise. PLoS ONE.

[24] Terauchi Y (1997). Development of non-insulin-dependent diabetes mellitus in the double knockout mice with disruption of insulin receptor substrate-1 and beta cell glucokinase genes. Genetic reconstitution of diabetes as a polygenic disease. J. Clin. Investig..

[25] Tamemoto H (1994). Insulin resistance and growth retardation in mice lacking insulin receptor substrate-1. Nature.

[26] Kubota N (2000). Disruption of insulin receptor substrate 2 causes type 2 diabetes because of liver insulin resistance and lack of compensatory beta-cell hyperplasia. Diabetes.

[27] Laustsen PG (2002). Lipoatrophic diabetes in Irs1(−/−)/Irs3(−/−) double knockout mice. *Genes &*. Development.

[28] Long YC, Cheng Z, Copps KD, White MF (2011). Insulin receptor substrates Irs1 and Irs2 coordinate skeletal muscle growth and metabolism via the Akt and AMPK pathways. Mol. Cell. Biol..

[29] Withers DJ (1998). Disruption of IRS-2 causes type 2 diabetes in mice. Nature.

[30] Liu F (2015). Upregulation of MG53 induces diabetic cardiomyopathy through transcriptional activation of peroxisome proliferation-activated receptor alpha. Circulation.

[31] Nguyen N, Yi JS, Park H, Lee JS, Ko YG (2014). Mitsugumin 53 (MG53) ligase ubiquitinates focal adhesion kinase during skeletal myogenesis. J. Biol. Chem..

[32] Li H (2015). Modulation of wound healing and scar formation by MG53 protein-mediated cell membrane repair. J. Biol. Chem..

[33] Clark LD, Clark RK, Heber-Katz E (1998). A new murine model for mammalian wound repair and regeneration. Clin. Immunol. Immunopathol..

[34] Ahn MK (2016). Mitsugumin 53 regulates extracellular Ca(2+) entry and intracellular Ca(2+) release via Orai1 and RyR1 in skeletal muscle. Sci. Rep..

[35] He B (2012). Enhancing muscle membrane repair by gene delivery of MG53 ameliorates muscular dystrophy and heart failure in delta-Sarcoglycan-deficient hamsters. Mol. Ther.: J. Am. Soc. Gene Ther..

[36] Ham YM, Mahoney SJ (2013). Compensation of the AKT signaling by ERK signaling in transgenic mice hearts overexpressing TRIM72. Exp. Cell Res..

[37] Zhang Y, Wu HK, Lv FX, Xiao RP (2016). MG53 is a double-edged sword for human diseases. Sheng li xue bao: [Acta physiologica Sin.].

[38] Wu HK (2019). Glucose-sensitive myokine/cardiokine MG53 Regulates systemic insulin response and metabolic homeostasis. Circulation.

[39] Zhu, H., Hsueh, W. & Whitson, B.A. Letter by Zhu et al. Regarding Article, “Glucose-Sensitive Myokine/Cardiokine MG53 Regulates Systemic Insulin Response and Metabolic Homeostasis”. *Circulation* **140**, e186-e187 (2019).​ 10.1161/CIRCULATIONAHA.118.039305 PMC9215306 31381425

[40] Zhu H (2011). Polymerase transcriptase release factor (PTRF) anchors MG53 protein to cell injury site for initiation of membrane repair. J. Biol. Chem..

