# Supplementary material for: Sustained elevation of MG53 in the bloodstream increases tissue regenerative capacity without compromising metabolic function
Source: Nat Commun. 2019 Oct 11;10:4659. doi: 10.1038/s41467-019-12483-0 (PMC6789113; doi:10.1038/s41467-019-12483-0)
Supplement: Supplementary file 1 — Supplementary Information [file 41467_2019_12483_MOESM1_ESM.pdf]

Supplementary Materials

for

Sustained Elevation of MG53 in the Bloodstream Increases Tissue Regenerative Capacity without Compromising Metabolic Function

Zehua Bian[1,*], Qiang Wang[1,*], Xinyu Zhou[1,*], Tao Tan[1], Ki Ho Park[1], H. Fritz Kramer[2], Alan McDougal[2], Nicholas J. Laping[3], Sanjay Kumar[3], T. M. Ayodele Adesanya[1], Matthew Sermersheim[1], Frank Yi[1], Xinxin Wang[4], Junwei Wu[4], Kristyn Gumpper[1], Qiwei Jiang[1], Duofen He[5], Pei-Hui Lin[1], Haichang Li[1], Fangxia Guan[6], Jingsong Zhou[7], Mark Kohr[8], Chunyu Zeng[5], Hua Zhu[1,¶] and Jianjie Ma[1,¶]

[1] Department of Surgery, Davis Heart and Lung Research Institute, The Ohio State University, Columbus, OH 43210
[2] Innate Immunity Research Unit, GlaxoSmithKline, Inc., Collegeville, PA 19426
[3] Novel Human Genetics Research Unit, GlaxoSmithKline, Inc., Collegeville, PA 19426
[4] The First Affiliated Hospital of Zhengzhou University, Zhengzhou 45000, Henan, China
[5] Department of Cardiology, Daping Hospital, The Third Military Medical University, Chongqing 400042, China
[6] School of Life Sciences, Zhengzhou University, Zhengzhou 450001, Henan, China
[7] College of Nursing and Health Innovation, University of Texas at Arlington, Arlington, TX 76019
[8] Department of Environmental Health and Engineering, Bloomberg School of Public Health, Johns Hopkins University, Baltimore, MD 21205

¶ Corresponding authors:

Jianjie Ma, Ph.D.

Tel. (614) 292-2636

Email: Jianjie.Ma@osumc.edu

Hua Zhu, Ph.D.

Tel. (614) 292-2130

Email: Hua.zhu@osumc.edu

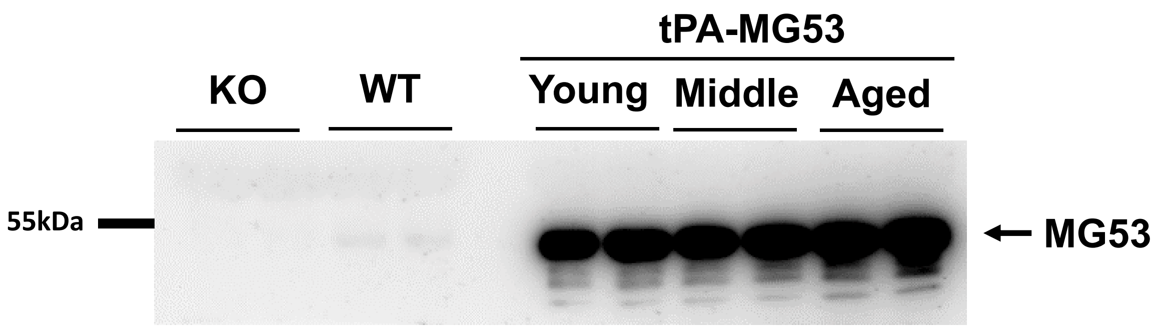

Supplementary Figure 1. tPA-MG53 mice maintain high expression of MG53 throughout the life span.

1 µl sera derived from 3-month *mg53-/-* (KO), wild type (WT) and tPA-MG53 mice at 2 month (young), 12 month (middle) and 24 month (aged) were probed with anti-MG53 antibody. The absence of MG53 signal in KO samples indicated specificity of antibody against MG53. In addition, circulating MG53 remained high in tPA-MG53 mice at different ages.

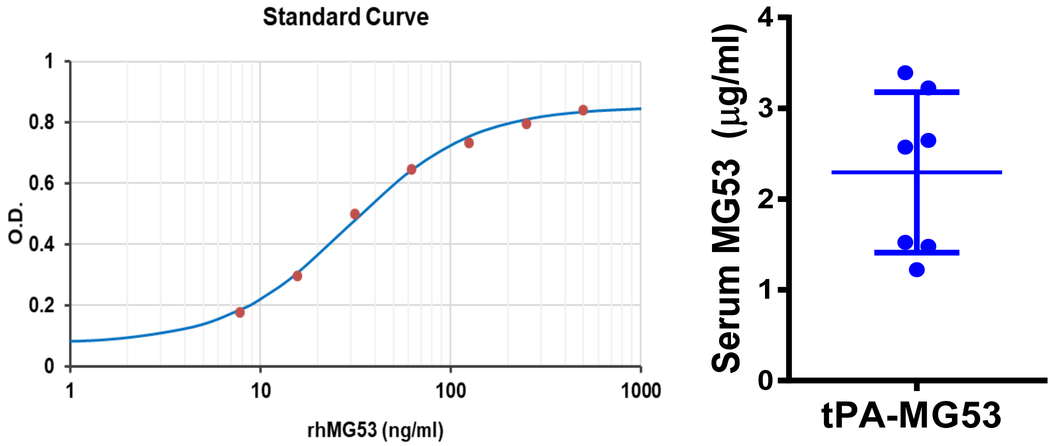

Supplementary Figure 2. ELISA determination of MG53 in sera derived from the tPA-MG53 mice.

A sandwich ELISA method was developed with two monoclonal antibodies (as capture and detection reagents). Different concentrations rhMG53 protein were spiked into the serum derived from *mg53-/-* mice to establish the standard curve for MG53 detection (*left panel*). This method has been used to measure the pharmacokinetic properties of intravenous administered rhMG53 protein in mouse, rats and dogs (Weisleder et al, 2012, Science Translational Medicine; Duann et al, 2015, Science Translational Medicine).

We used this ELISA to determine the sera levels of MG53 from the tPA-MG53 mice (*right panel*). Serum levels of MG53 in the wild type mice is beyond the detection limit of the ELISA. Error bar represents the standard deviation.

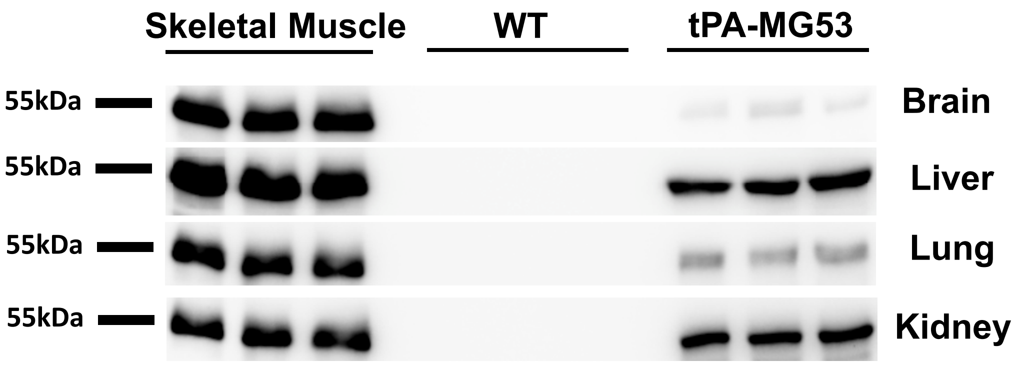

Supplementary Figure 3. Expression of MG53 in non-muscle tissues derived from the tPA-MG53 mice.

For comparison of the protein level of MG53 present in non-muscle tissues in the tPA-MG53 mice, western blots were conducted with loading of 2 µg skeletal muscle (left 3 lanes), and 20 µg of other tissues in the same gel. Enhanced western blot pictures were used to show the presence of MG53 in brain, kidney, lung and liver tissues derived from the tPA-MG53 mice. The data showed that non-muscle tissues also contain increased level of MG53 in the tPA-MG53 mice compared with the wild type tissues. The amount of MG53 present in the non-muscle tissues is ~1-5% of that present in skeletal muscle, which could reflect uptake of MG53 protein from circulation.

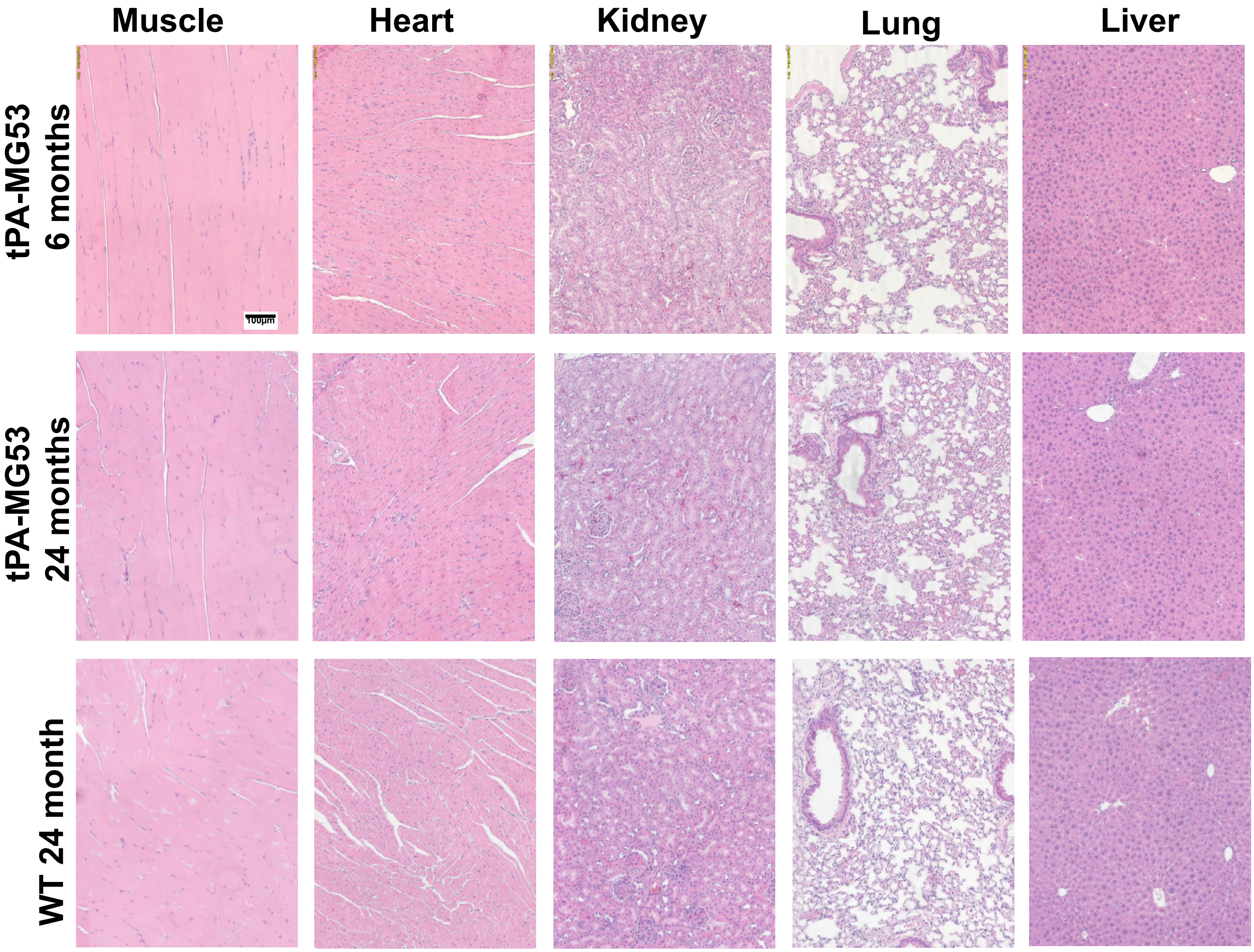

Supplementary Figure 4. H/E staining of major vital organs with WT and tPA-MG53 mice at different ages.

Representative H/E staining of muscle, heart, lung, kidney, and liver tissues derived from the tPA-MG53 mice at 6 months (upper panels), 24 months of age (middle panels) and wild type mice at 24 months of age (lower panels). No apparent pathologies were identified.

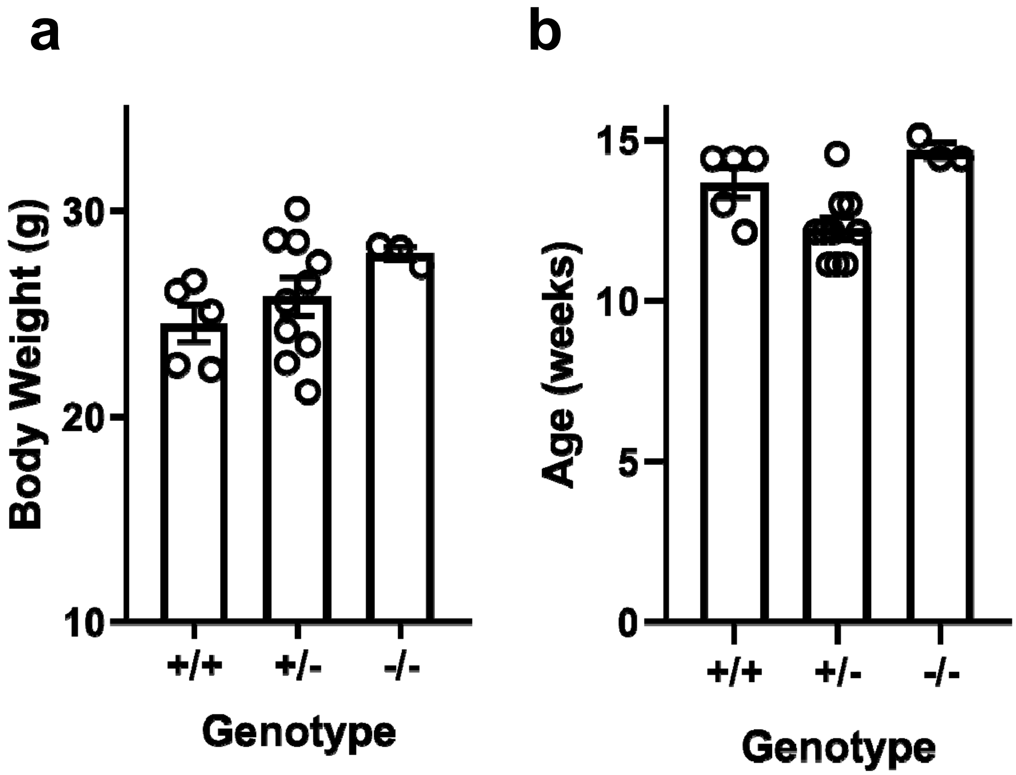

Supplementary Figure 5. Body weight measurements from WT (+/+), and MG53 heterozygous (+/-) and homozygous (-/-) knockout littermate mice. Male mice were maintained in a mixed genetic background on a standard chow diet. **a)** Body weight was assessed between 11 to 15 weeks of age (n = 3-10 mice/group). **b)** Age at the time of body weight measurement. Error bar represents the standard deviation.

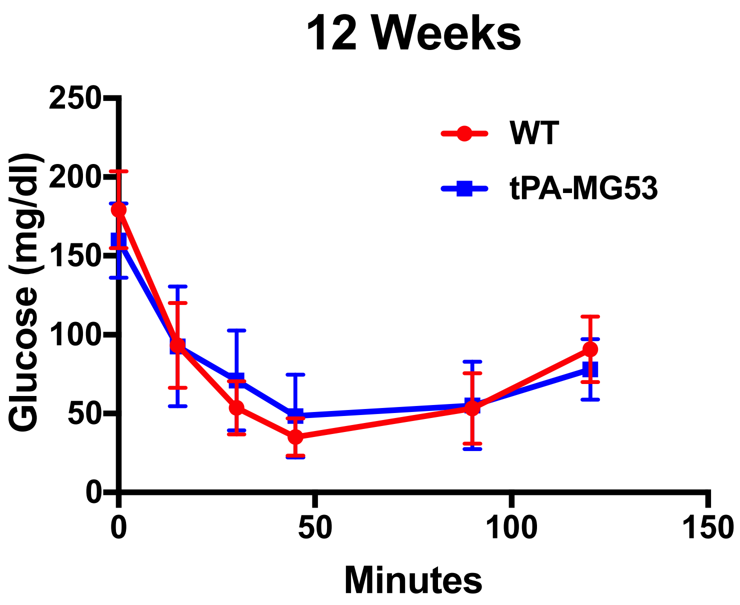

Supplementary Figure 6. tPA-MG53 and wild type littermates show similar response to insulin at age of 12 weeks.

Insulin-tolerance tests were conducted with tPA-MG53 and WT littermates at the age of 12 weeks. n=6 per group. Error bar represents the standard deviation.

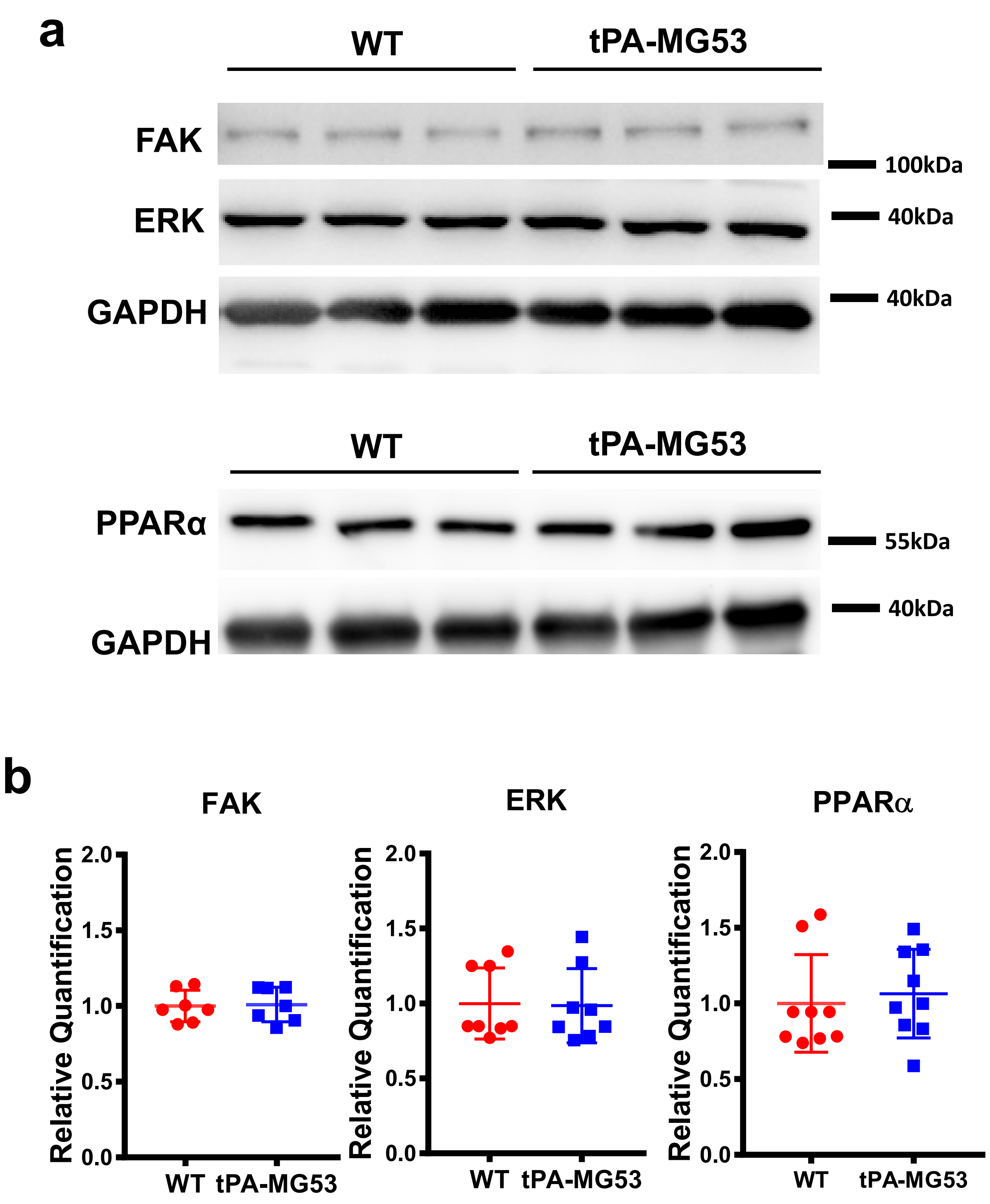

Supplementary Figure 7. Western blot of FAK, ERK and PPARα expression in skeletal muscle derived from wild type and tPA-MG53 mice.

Western blot of tibialis muscle derived from WT and tPA-MG53 mice at 6 months of age. Quantification of FAK, ERK and PPARα showed no significant difference between the two groups (N=7-9/group). Error bar represents the standard deviation.

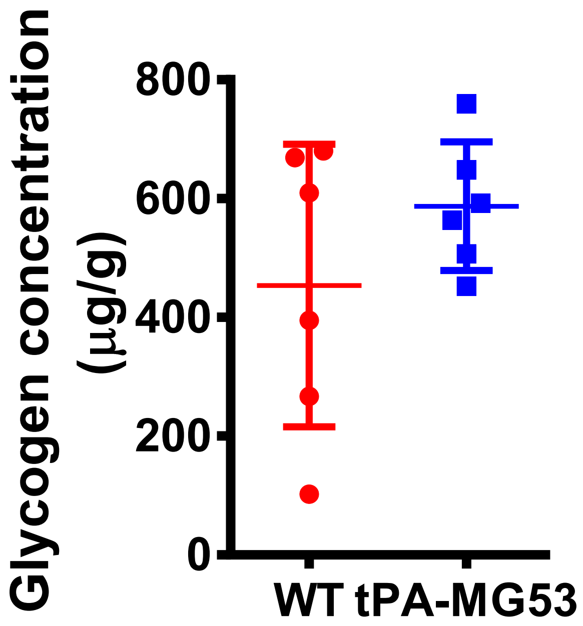

Supplementary Figure 8. tPA-MG53 muscles have similar glycogen content as that in wild type muscles.

Biochemical analysis of glycogen content was measured in gastrocnemic muscle derived from WT and tPA-MG53 mice at 6 months of age. Quantification of glycogen concentration showed a trend for a higher amount in the tPA-MG53 muscle, but the P value does not support statistical significant difference (n=6 per group, P = 0.2398). Error bar represents the standard deviation.

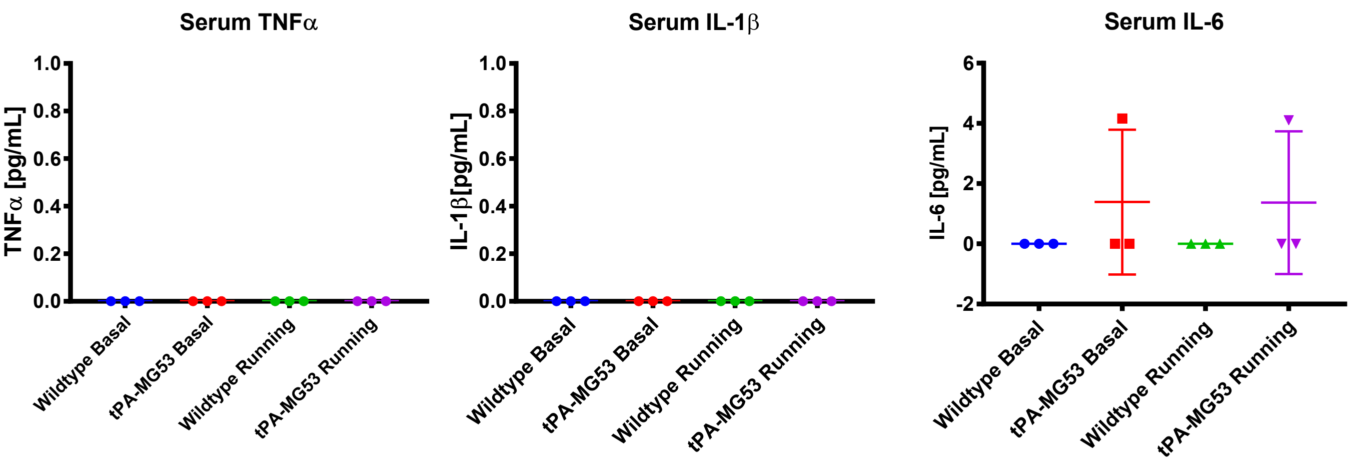

Supplementary Figure 9. Voluntary wheel running does not stimulate inflammatory cytokine release from wild type and tPA-MG53 mice.

ELISA analysis of TNF-α, IL-1β and IL-6 were performed in serum samples derived from wild type and tPA-MG53 mice. Quantification of these cytokines showed no elevation after 7 day voluntary wheel running. n=3 per group. Error bar represents the standard deviation.

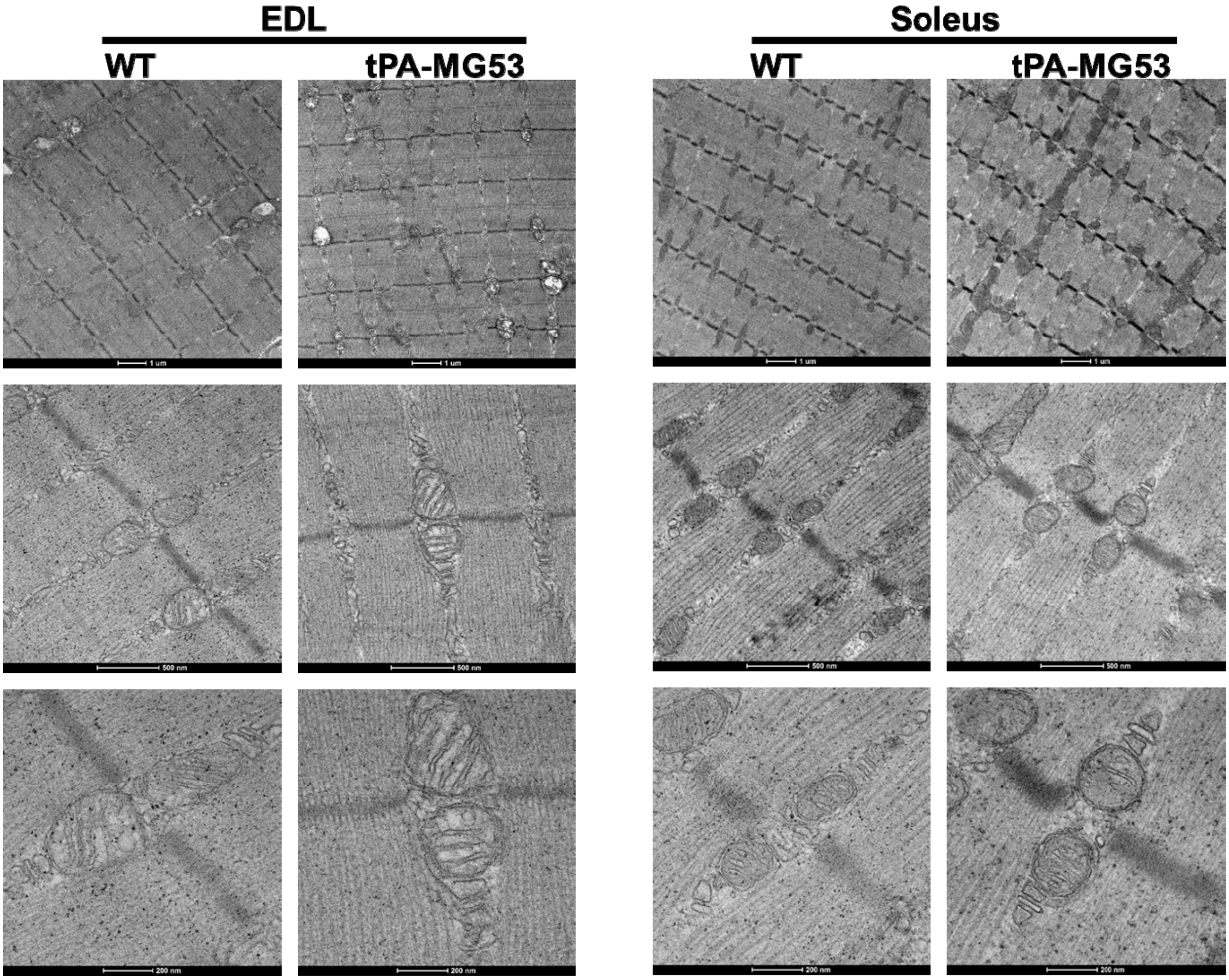

Supplementary Figure 10. Mitochondria morphology in wild type and tPA-MG53 muscle remains similar.

Both EDL (Left) and Soleus (Right) muscle derived from wild type and tPA-MG53 were subjected to EM analysis for mitochondria morphology. EM pictures revealed no difference of morphology of mitochondria derived from wild type and tPA-MG53 muscle.

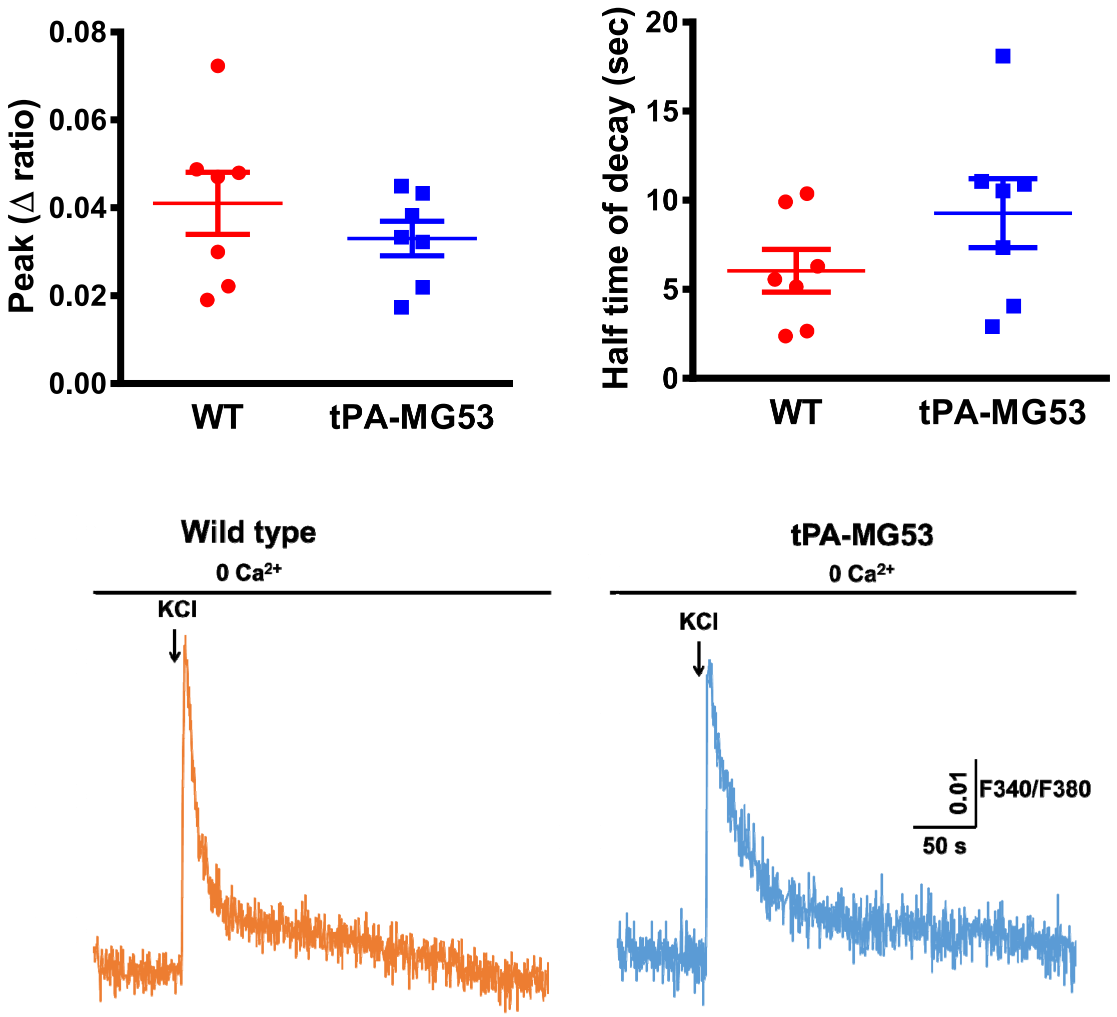

Supplementary Figure 11. KCl induced intracellular Ca release in FDB fibers derived from wild type and tPA-MG53 mice.

Both Ca peak value (upper left panel) and half time of decay (upper right panel) remain similar in wild type and tPA-MG53 muscle fibers when the extracellular solution contained 0 Ca^{2+}. Representative Ca^{2+} trace of wild type and tPA-MG53 fibers are shown in lower panels. Error bar represents the standard deviation.

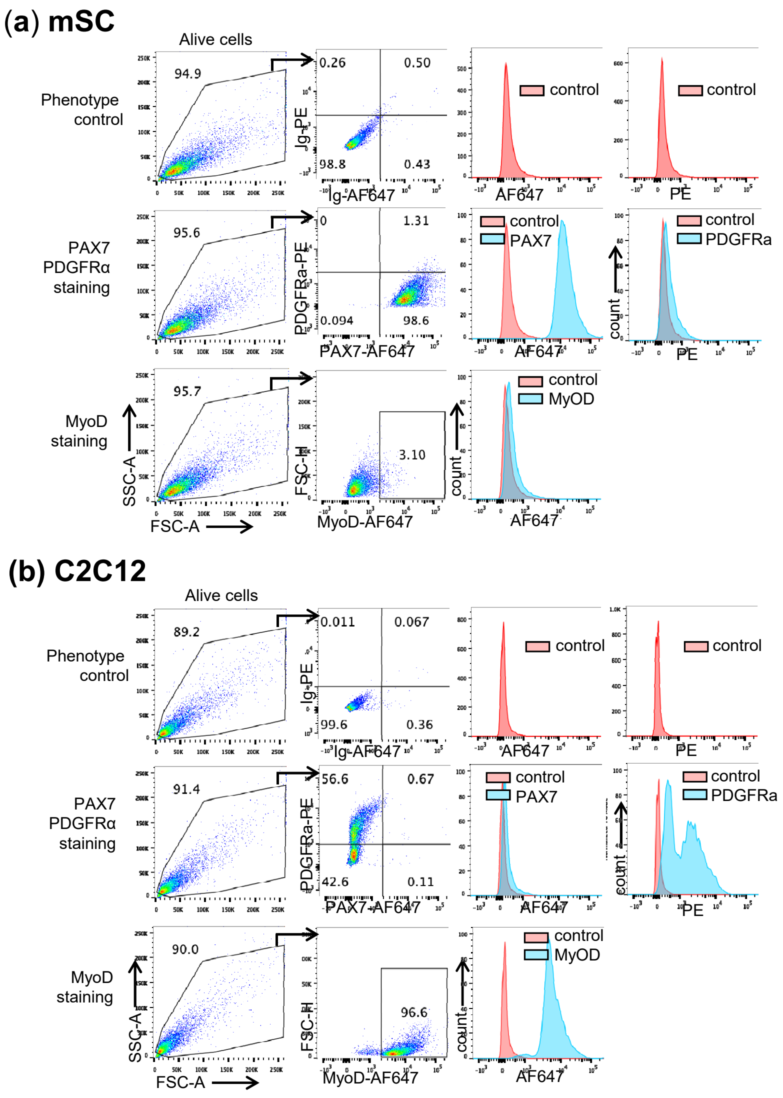

Supplementary Figure 12. Graphically account for all FACS sequential gating/sorting strategies for Figure 6b. (a) Muscle satellite cell (mSC) were stainined with antibodies against Pax 7, PDGFRα and MyoD. PE or AF647 Ig was used for phenotype control. **(b)** Similarly, C2C12 cells were stainined with antibodies against Pax 7, PDGFRα and MyoD. PE or AF647 Ig was used for phenotype control.

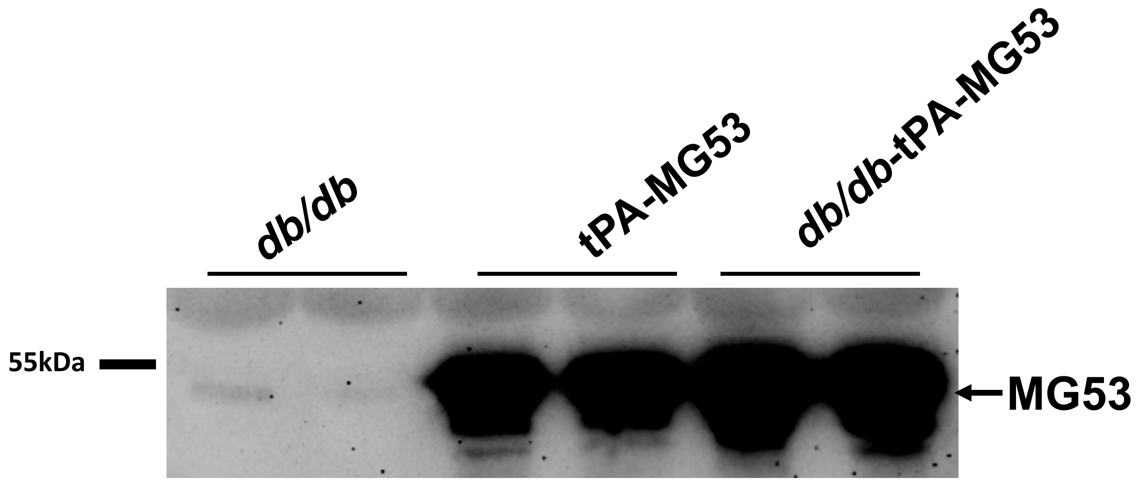

Supplementary Figure 13. Western blot of MG53 expression in sera derived from *db/db*, tPA-MG53 and *db/db*-tPA-MG53 mice.

1 μl of serum was loaded per lane. Littermate mice at age of 32 weeks were used.

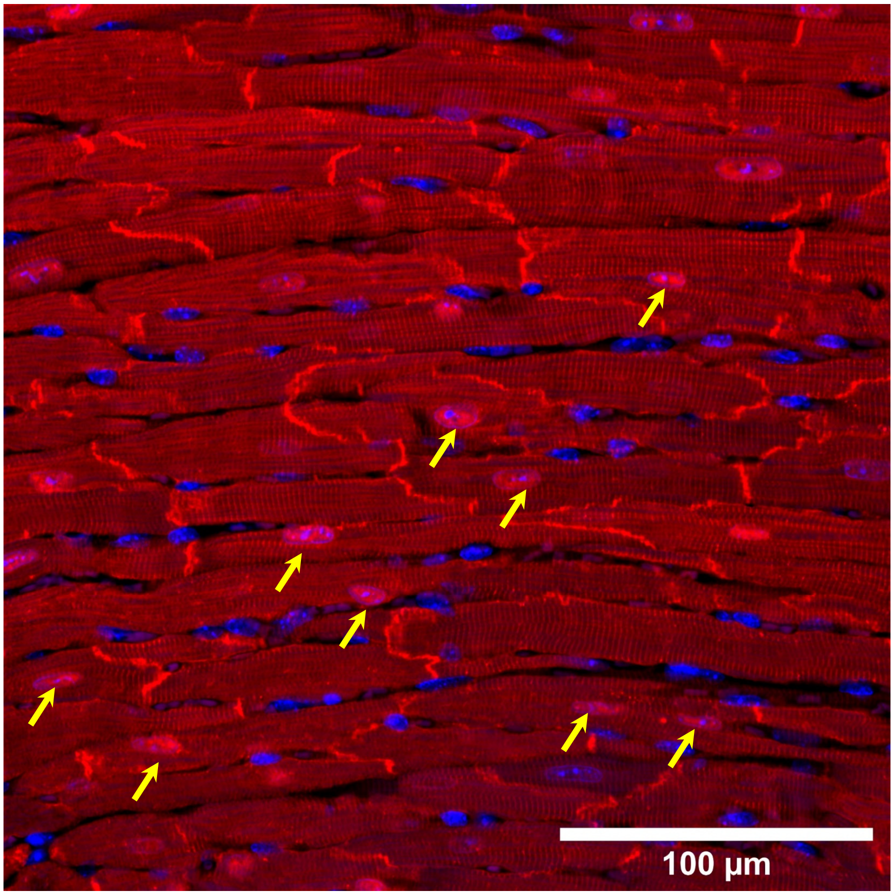

Supplementary Figure 14. Immunohistochemical staining of cardiomyocytes derived from the tPA-MG53 mouse heart showed nuclear localization of MG53 (yellow arrows). Red fluorescence represents staining with anti-MG53 antibody. Blue represents DAPI staining of nucleus.
