# Supplementary material for: Sustained elevation of MG53 in the bloodstream increases tissue regenerative capacity without compromising metabolic function
Source: Nat Commun. 2019 Oct 11;10:4659. doi: 10.1038/s41467-019-12483-0 (PMC6789113; doi:10.1038/s41467-019-12483-0)
Supplement: Supplementary file 3 — Description of Additional Supplementary Files [file 41467_2019_12483_MOESM3_ESM.pdf]

Description of Additional Supplementary Files

File Name: Supplementary Movie 1

Description: **tPA-MG53 mice at age of 32 months ran better than wild type mice at age of 6 months.** tPA-MG53 mice (32 month) and wild type mice (6 month) have been running on treadmill for 1 Hour. The movie was taken at the end of 1 Hour running at 10 meter/min speed.
