# Supplementary material for: Sustained elevation of MG53 in the bloodstream increases tissue regenerative capacity without compromising metabolic function
Source: Nat Commun. 2019 Oct 11;10:4659. doi: 10.1038/s41467-019-12483-0 (PMC6789113; doi:10.1038/s41467-019-12483-0)
Supplement: Supplementary file 5 — Reporting Summary [file 41467_2019_12483_MOESM5_ESM.pdf]

nature research

Corresponding author(s): Jianjie Ma and Hua Zhu

Reporting Summary

Nature Research wishes to improve the reproducibility of the work that we publish. This form provides structure for consistency and transparency in reporting. For further information on Nature Research policies, see Authors & Referees and the Editorial Policy Checklist.

Statistical parameters

When statistical analyses are reported, confirm that the following items are present in the relevant location (e.g. figure legend, table legend, main text, or Methods section).

n/a	Confirmed	
☐	☒	The exact sample size (n) for each experimental group/condition, given as a discrete number and unit of measurement
☐	☒	An indication of whether measurements were taken from distinct samples or whether the same sample was measured repeatedly
☐	☒	The statistical test(s) used AND whether they are one- or two-sided<br>*Only common tests should be described solely by name; describe more complex techniques in the Methods section.*
☐	☒	A description of all covariates tested
☐	☒	A description of any assumptions or corrections, such as tests of normality and adjustment for multiple comparisons
☐	☒	A full description of the statistics including central tendency (e.g. means) or other basic estimates (e.g. regression coefficient) AND variation (e.g. standard deviation) or associated estimates of uncertainty (e.g. confidence intervals)
☐	☒	For null hypothesis testing, the test statistic (e.g. F, t, r) with confidence intervals, effect sizes, degrees of freedom and P value noted<br>*Give P values as exact values whenever suitable.*
☒	☐	For Bayesian analysis, information on the choice of priors and Markov chain Monte Carlo settings
☒	☐	For hierarchical and complex designs, identification of the appropriate level for tests and full reporting of outcomes
☒	☐	Estimates of effect sizes (e.g. Cohen's d, Pearson's r), indicating how they were calculated
☐	☒	Clearly defined error bars<br>*State explicitly what error bars represent (e.g. SD, SE, CI)*

Our web collection on statistics for biologists may be useful.

Software and code

Policy information about availability of computer code

Data collection	ImageJ 1.49m
Data analysis	Graphpad Prism 5

For manuscripts utilizing custom algorithms or software that are central to the research but not yet described in published literature, software must be made available to editors/reviewers upon request. We strongly encourage code deposition in a community repository (e.g. GitHub). See the Nature Research guidelines for submitting code & software for further information.

Data

Policy information about availability of data

All manuscripts must include a data availability statement. This statement should provide the following information, where applicable:

- Accession codes, unique identifiers, or web links for publicly available datasets
- A list of figures that have associated raw data
- A description of any restrictions on data availability

The authors declare that all data supporting the findings of this study are available within the paper.

Field-specific reporting

Please select the best fit for your research. If you are not sure, read the appropriate sections before making your selection.

☒ Life sciences ☐ Behavioural & social sciences ☐ Ecological, evolutionary & environmental sciences

For a reference copy of the document with all sections, see nature.com/authors/policies/ReportingSummary-flat.pdf

Life sciences study design

All studies must disclose on these points even when the disclosure is negative.

Sample size	For our in vivo experiments, we chose n=5-10 animals per group. We didn't use software to determine the sample size. We chose this sample size based on our previous experiences.
Data exclusions	No date were excluded.
Replication	The biochemical and molecular biology experiments in this paper have been repeated at least 2 times (total 3 independent experiments). We didn't notice failure of the replication experiments.
Randomization	For experiments, including GTT, ITT, ear punch, treadmill running and cardiotoxin injury experiments, we randomly treated the mice with different genotypes.
Blinding	For experiments, including GTT, ITT, ear punch, treadmill running and cardiotoxin injury experiments, the operators were blinded with mouse genotypes. In all subsequent histologic analyses, the individual was masked to the genotypes.

Reporting for specific materials, systems and methods

Materials & experimental systems

n/a	Involved in the study	
☒	☐	Unique biological materials
☐	☒	Antibodies
☒	☐	Eukaryotic cell lines
☒	☐	Palaeontology
☐	☒	Animals and other organisms
☒	☐	Human research participants

Methods

n/a	Involved in the study	
☒	☐	ChIP-seq
☐	☒	Flow cytometry
☒	☐	MRI-based neuroimaging

Antibodies

Antibodies used	Custom-made monoclonal anti-MG53 antibody or commercial IRS-1 antibody (Invitrogen, Cat. No. 700662), anti-PPARα antibody (Novus Bio, Cat. No. NB600-636), anti-IRβ antibody (Cell Signaling, Cat. No. 3025), anti-Glut-4 antibody (Cell Signaling, Cat. No. 2213), and anti-GAPDH antibody (Cell Signaling Technology, Cat. No. 2118s), anti-MHC I antibody (DSHB, Cat. No. BA-F8), anti-MHC IIa (DSHB, Cat. No. SC-71), anti-MHC IIb (DSHB, Cat. No. BF-F3), anti-MHC IIx (DSHB, Cat. No. 6H1) were used in this study.
Validation	Custom-made anti-MG53 antibody is validated with tissues from WT and mg53-/- tissues. This antibody has also been used and validated by other investigators. We validated the antibodies based on molecular weight, reports on the manufacture websites and published research articles that cited the same source of the antibodies as we used in our study.

Animals and other organisms

Policy information about studies involving animals; ARRIVE guidelines recommended for reporting animal research

Laboratory animals	Male and female mg53-/- mice and their wild type littermates, male and female tPA-MG53 mice and their wild type littermates were used for the experiments.
Wild animals	the study did not use wild animals.
Field-collected samples	This study did not use samples collected in the field.

Flow Cytometry

Plots

Confirm that:

☒ The axis labels state the marker and fluorochrome used (e.g. CD4-FITC).

☒ The axis scales are clearly visible. Include numbers along axes only for bottom left plot of group (a 'group' is an analysis of identical markers).

☒ All plots are contour plots with outliers or pseudocolor plots.

☒ A numerical value for number of cells or percentage (with statistics) is provided.

Methodology

Sample preparation	The samples used in the manuscript were primary cultured muscle satellite cells (from single mouse skeletal msucle fibers) and C2C12 cell line. Cells were labeled with monoclonal or polyclonal antibodies at 4-degree temperature for 30 minutes and washed with PBS containing 2% BSA prior to analysis using an LSRII flow cytometer (BD Biosciences) to detect expression of each antigen.
Instrument	LSRII flow cytometer (BD Biosciences)
Software	Data were collected using FACSDiva (BD) and analyzed using FACSDiva (BD) or FlowJo v7.6 or v10. Graphing and statistical analysis were performed using Prism 5 (GraphPad).
Cell population abundance	We did not sort our cells in our experiments.
Gating strategy	Unless otherwise indicated, positive and negative gates were set using fluorophore-matched IgG controls as described in sample preparation. All refer the supplementary figure 12.

☒ Tick this box to confirm that a figure exemplifying the gating strategy is provided in the Supplementary Information.
