# Supplementary material for: Sustained elevation of MG53 in the bloodstream increases tissue regenerative capacity without compromising metabolic function
Source: Nat Commun. 2019 Oct 11;10:4659. doi: 10.1038/s41467-019-12483-0 (PMC6789113; doi:10.1038/s41467-019-12483-0)

Fig 1a

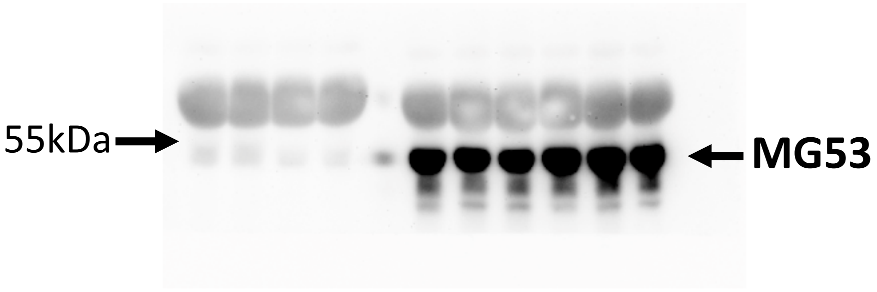

Fig 1c

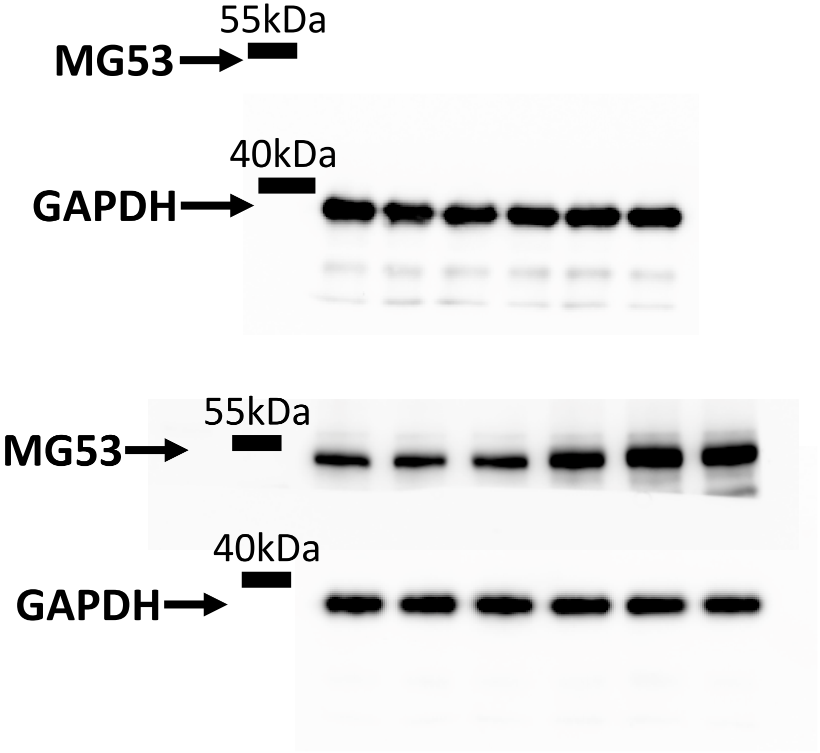

Fig 2d

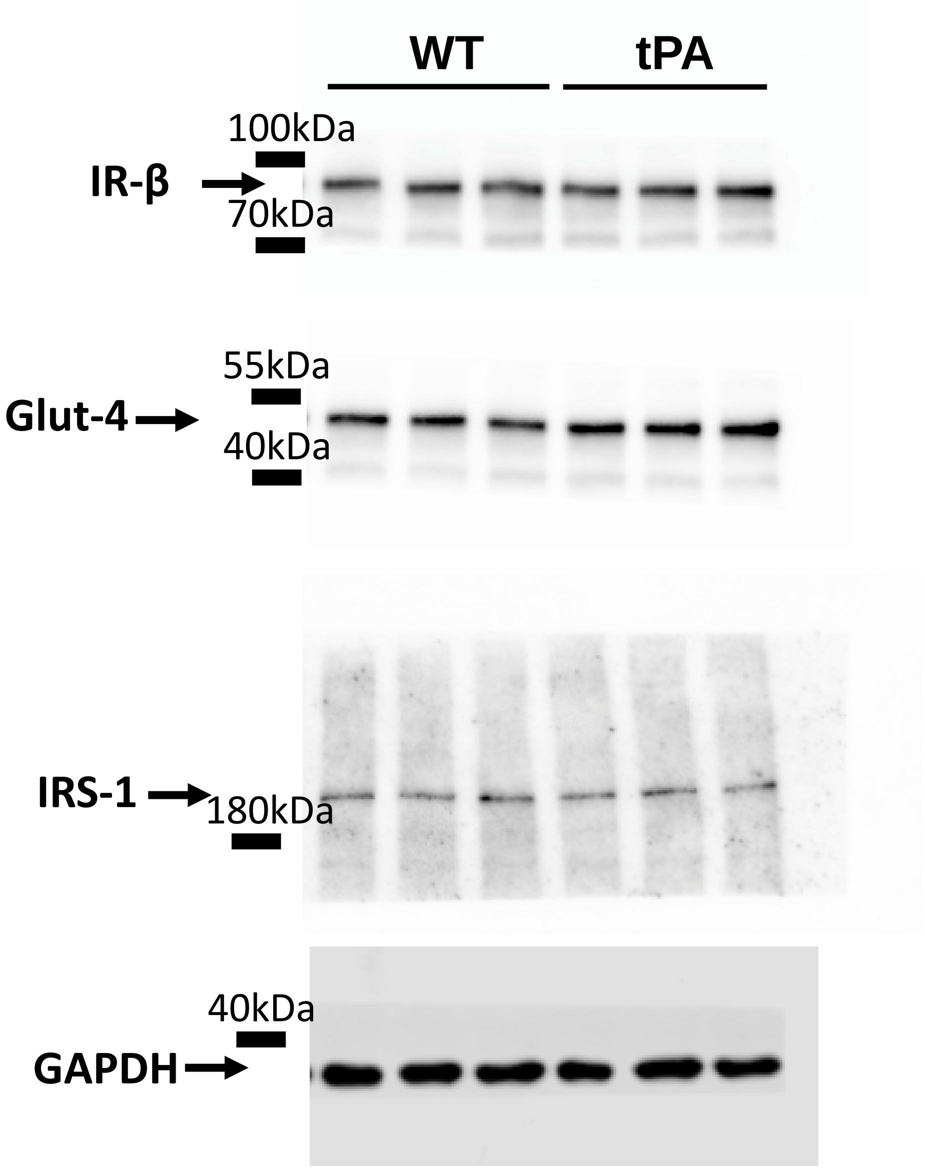
WT
tPA
100kDa
IR-β
70kDa
55kDa
Glut-4
40kDa
IRS-1
180kDa
40kDa
GAPDH

Fig 2f

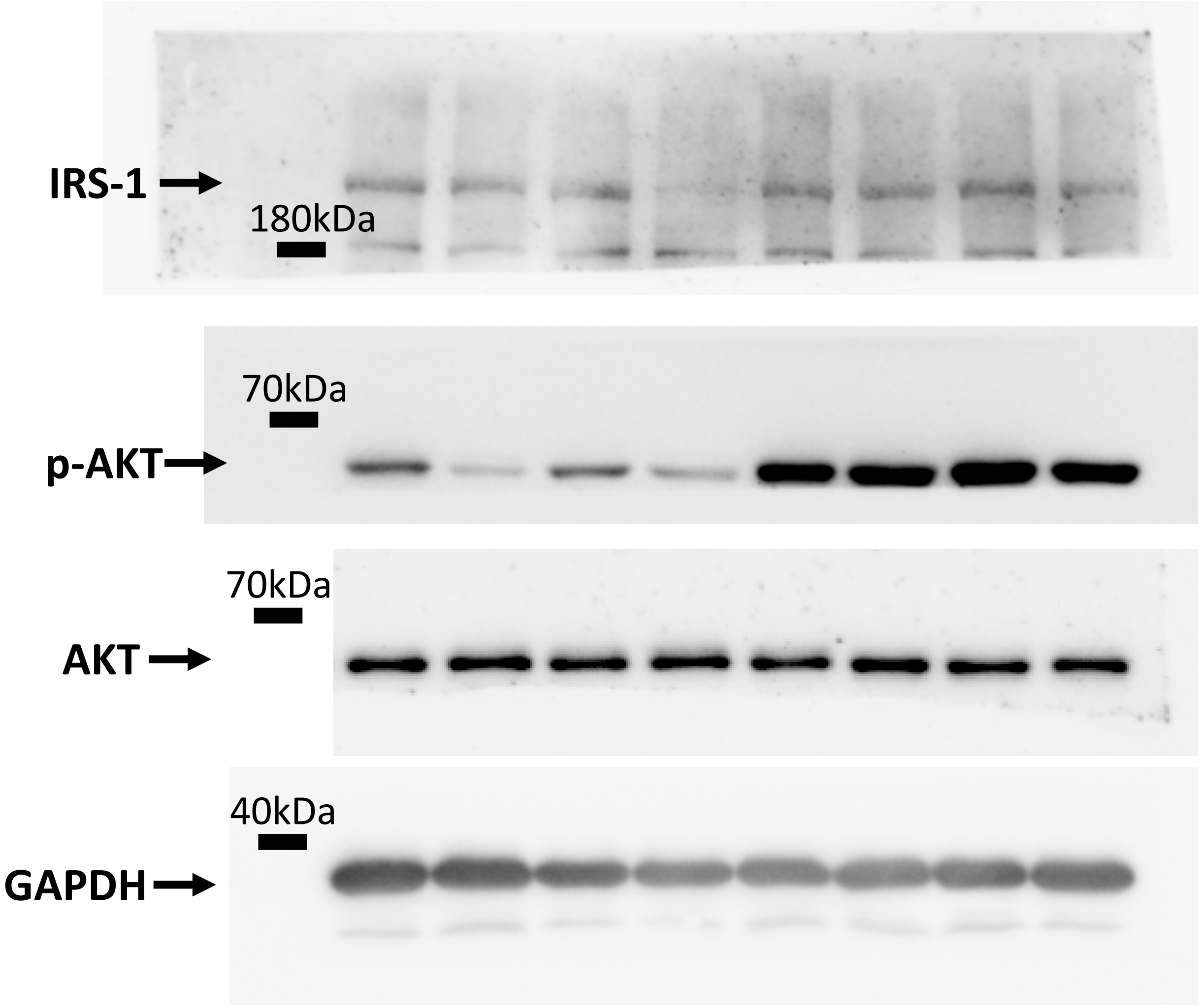
IRS-1
180kDa
70kDa
p-AKT
70kDa
AKT
40kDa
GAPDH

Fig 3a

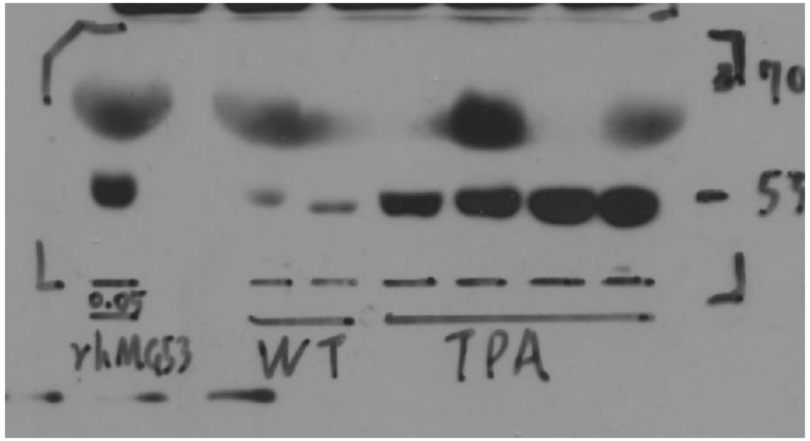

Fig 8d

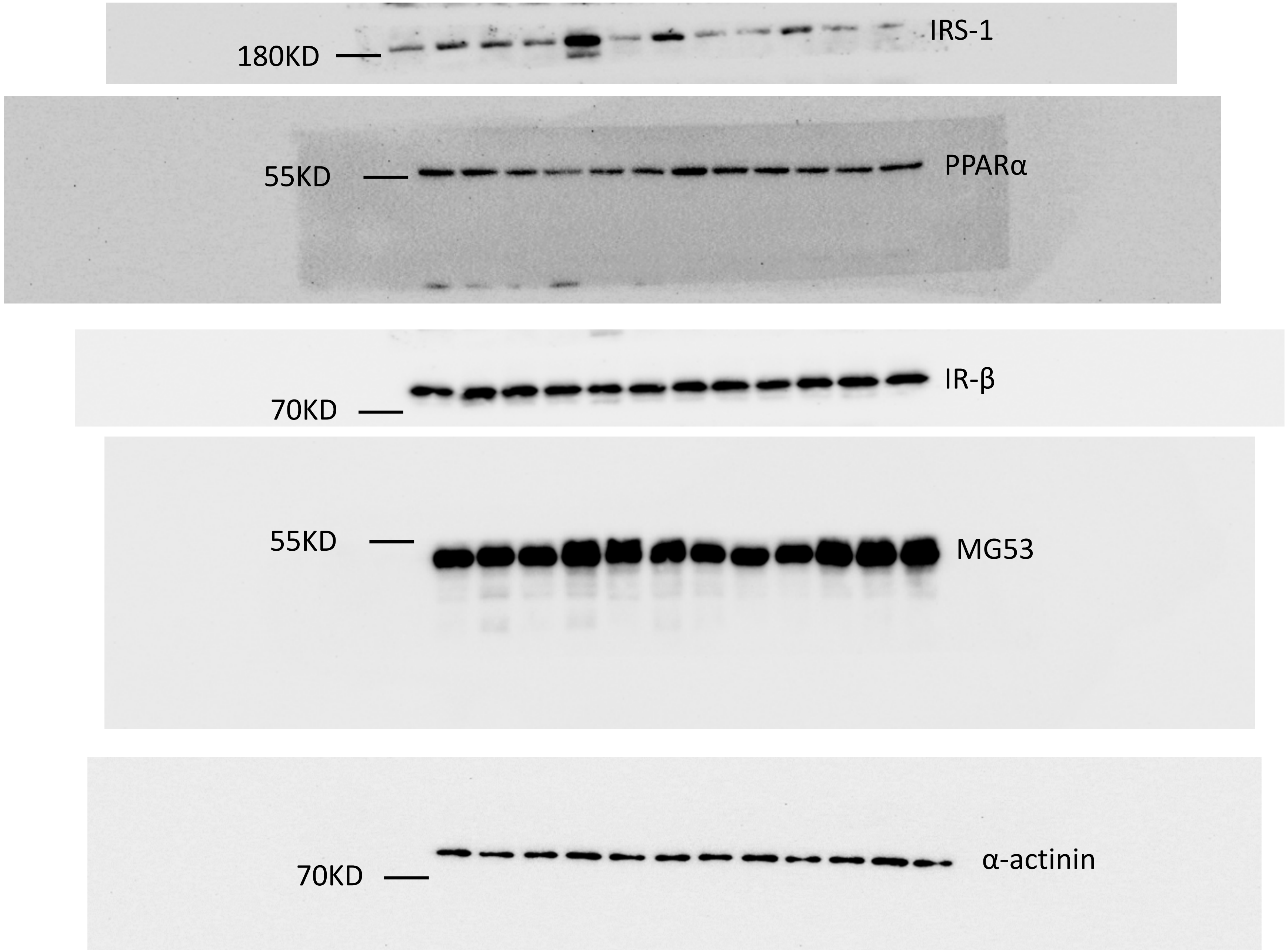

Supplemental Fig. S1

MG53 → 55kDa →

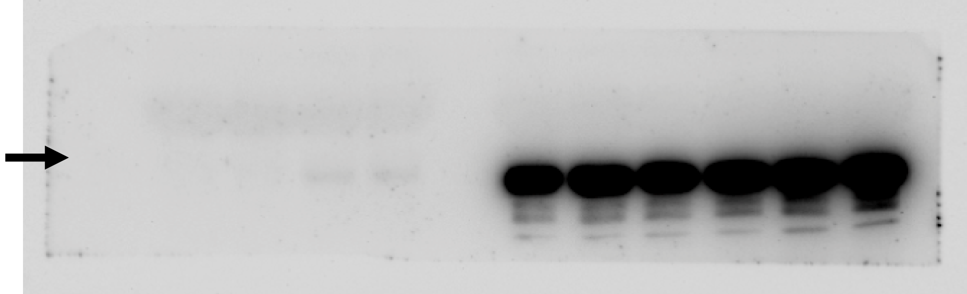

Supplemental Fig. S3

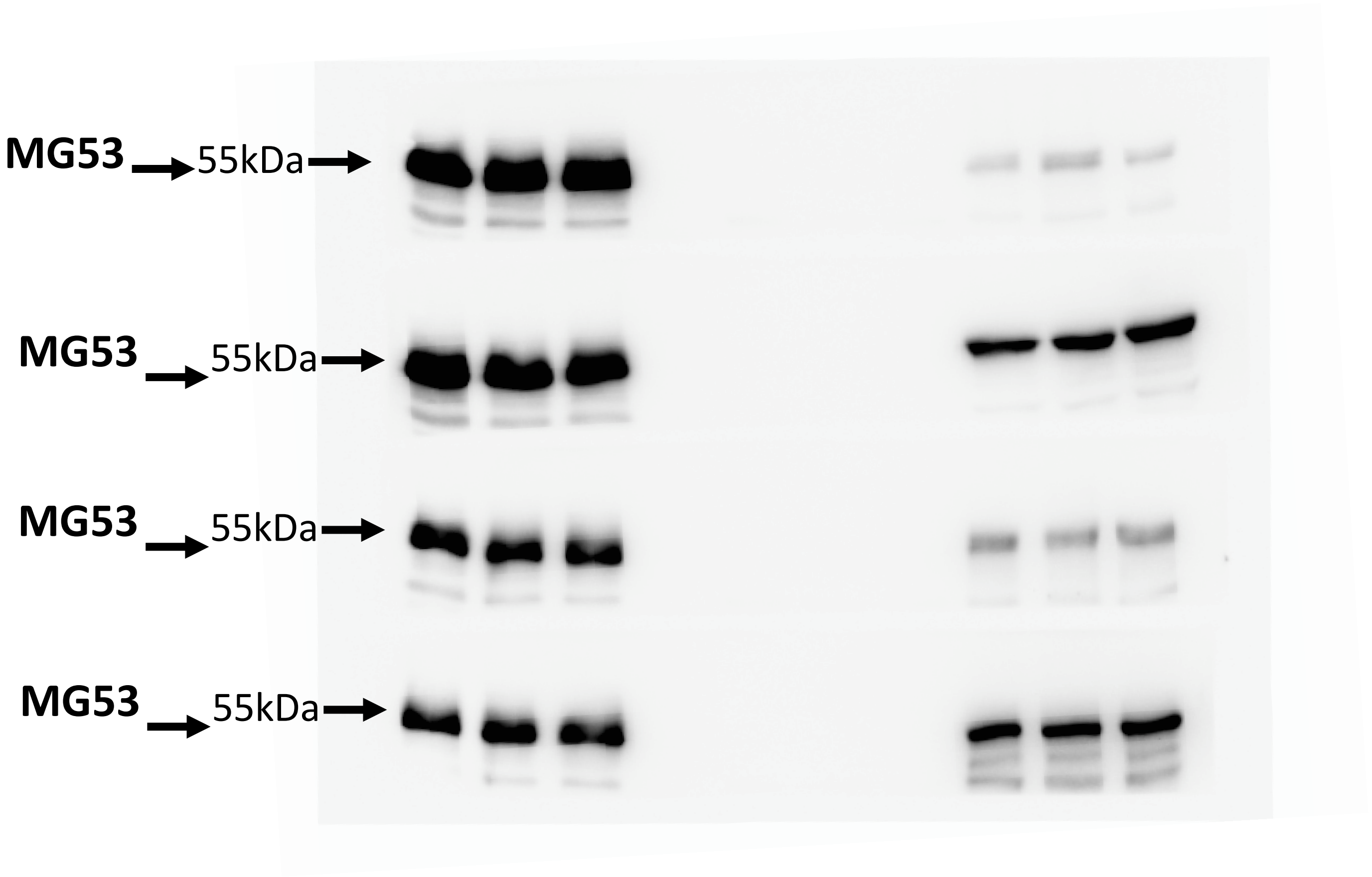

Supplemental Fig. S7

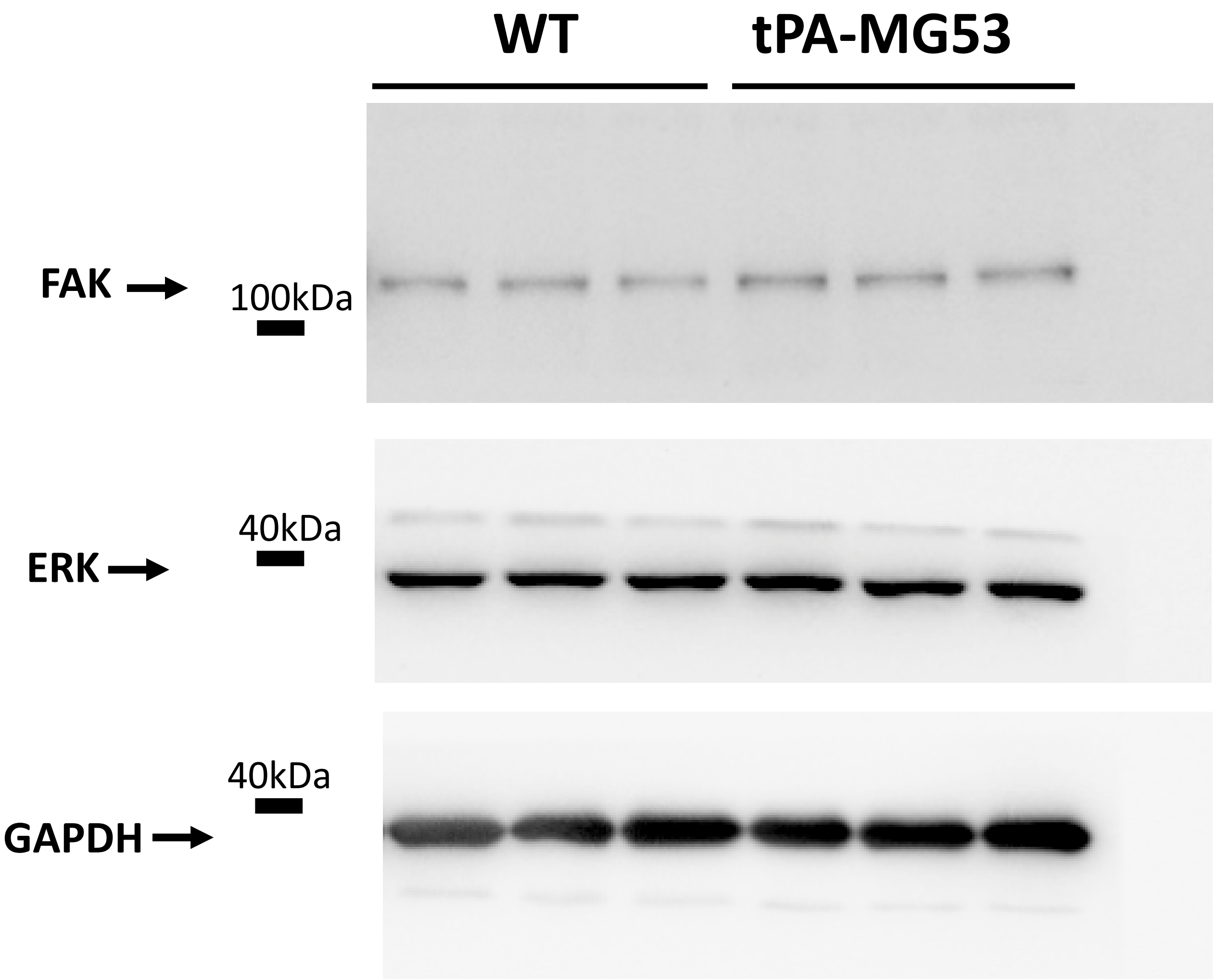

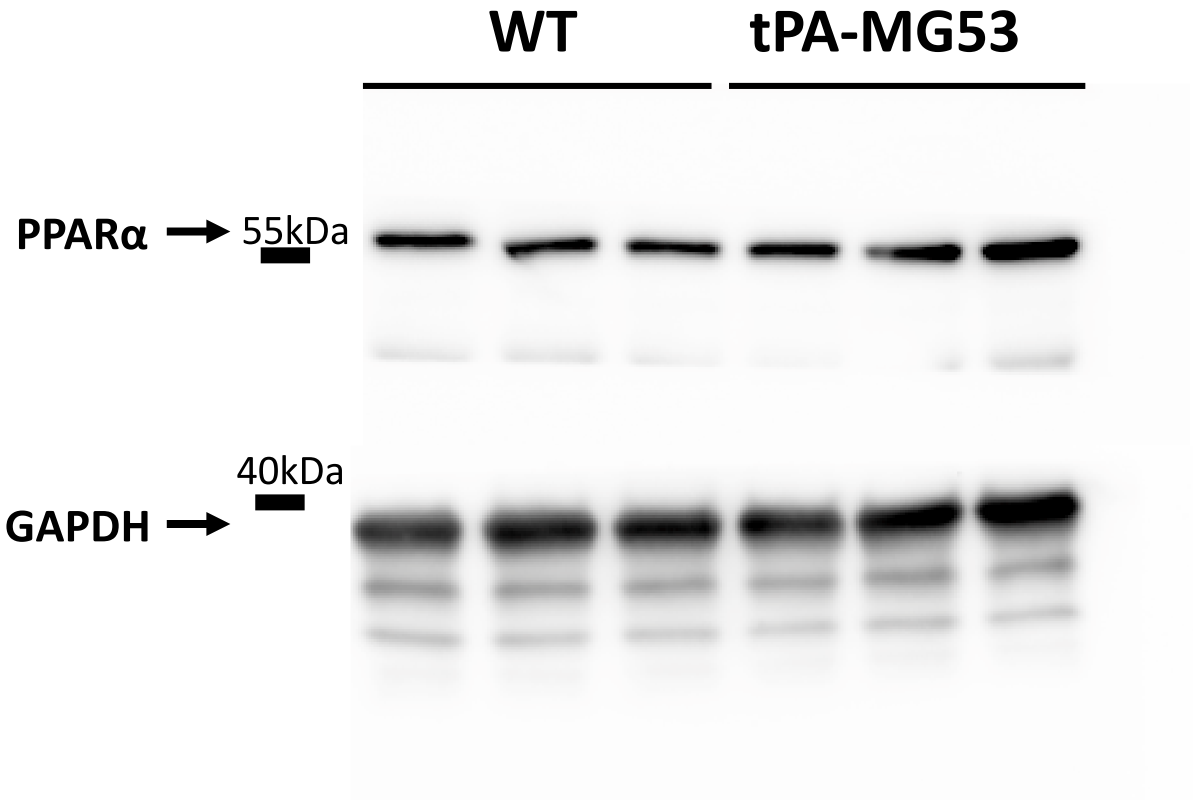

Supplemental Fig. S12

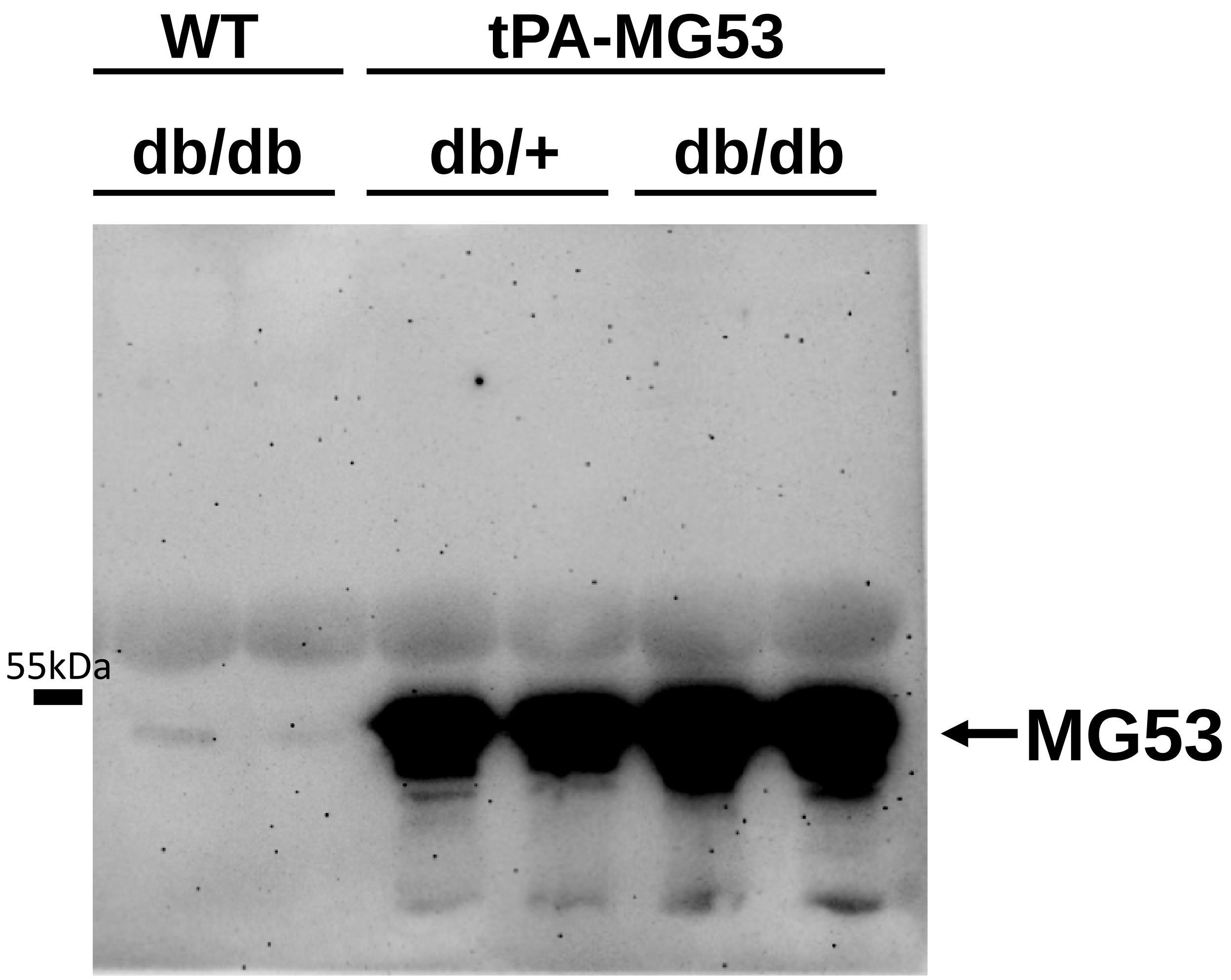

Supplement: Supplementary file 6 — Source Data [file 41467_2019_12483_MOESM6_ESM.zip › NCOMMS-18-32280 07-21-2019 Western Blots.pdf]
