# Supplementary material for: Sustained elevation of MG53 in the bloodstream increases tissue regenerative capacity without compromising metabolic function
Source: Nat Commun. 2019 Oct 11;10:4659. doi: 10.1038/s41467-019-12483-0 (PMC6789113; doi:10.1038/s41467-019-12483-0)
Supplement: Supplementary file 2 — Peer Review [file 41467_2019_12483_MOESM2_ESM.pdf]

Reviewers' comments:

Reviewer #1 (Remarks to the Author):

The authors state that the presence of high levels of circulating MG53 in blood increase tissue regeneration capacity. They test muscle regeneration after cardiotoxin injection and skin regeneration in lesions produced in the ear of the mice. For this purpose they use a transgenic mouse line tPA-MG53 that produces sustained expression of the protein in peripheral blood. The authors state that after 36 months they did not observe side effects on metabolic function. Finally, they claim that sustained overexpression of MG53 in circulation is effective to treat acute muscle tissue injuries and dermal wounds.

The paper is interesting and may have therapeutical implications.

However there are some weak points.

I agree that cardiotoxin injury is a common method to assay muscle regeneration in mice but it is far from the muscle injury that is commonly found in patients or in healthy patients suffering sport injuries for instance. I believe that other models such as treadmill exercise would be more realistic. It would be also interesting to cross the tPA-MG53 with a muscular dystrophy model in a way similar to what the authors did with de db/deb mouse to demonstrate a lack of effect in of sustained circulating MG53 on mouse metabolism. Even in acute injury the amount of recombinant MG53 necessary to observe an effect in a hypothetic trial in humans can not be predicted from the experiments performed since the transgenic models produces a sustained and massive expression of MG53 in plasma.

I think the paper is acceptable as a proof of principle but not suitable for a journal such as Nature Communications.

Reviewer #2 (Remarks to the Author):

This paper describes a new mouse model overexpressing MG53 in circulation, which have markedly improved injury repair and regeneration capacity. Overall, I think this paper has some very interesting and novel findings. However, I do have some issues with the manuscript in its present form and I thinks some of the experiments/measurements lack power or could have been performed in a more optimal way to support the conclusions.

Major:

o For Fig 2C+D, there seems to be a tendency towards increased insulin action in the MG53 mice and the error-bars are huge with n=5-6. Suggest to add more mice to show convincingly whether the difference is there or not. If different, the reason for the difference should be ascertained. Is insulin-signaling increased in different metabolic tissues? Is glucose uptake? Do the mice then secrete less insulin during the GTT to explain the lack of difference in GTT?

o For the running performance data in Fig 4E, the interpretation is that the recovery from prior exercise is improved in MG53 mice. Was the running performance in response to a single bout of acute exercise the same or is this phenotype only seen with repeated bouts, i.e. is there a difference in base-line exercise capacity? Is the phenotype muscle-injury related (inflammatory markers, myopathy markers, injury markers) or is this a metabolic effect (mitochondrial proteins, glucose and fat metabolism protein markers)? Is this also a phenotype seen with less extreme exercise regimens? Is exercise-induced substrate utilization changed? At the very least, I suggest to quantify the acute exercise capacity of these mice using a conventional incremental ramp test and perform some of the same analyses as in Fig 5 at the time where you observe the reduced running capacity. The n in this experiment is not stated in the legend.

o You state in the abstract that the MG53 mice live a healthier and longer life-span. This seems to be based on the observation in the results that 3 of 5 mice survived over 36 months whereas all WT mice died below 30 mo. How many mice in the WT group and was this significant? Technically, I acknowledge that the statement regarding life-span is correct but n=5 seems a bit low for life-span analysis (or any other kind). In terms of health, you seem to refer to Fig 1D and S3 as evidence for a healthy life span with no observable pathology. However, there seem to be no control mice quantified making it very difficult to judge how healthy the phenotype looks. Also, how many mice are the shown stainings representative of?

o You state that the MG53 mice have normal insulin signalling. This seems to refer to the total protein blots in Fig 2. You should run a +/- insulin-stimulation setup, e.g. your ITT, take tissues and measure some insulin-responsive phosphorylations or modify these statement.

Minor:

o Representative western blots provide 2 important pieces of information to the reader. 1) Is the signal/noise ratio reasonable 2) Does the band run at the correct MW. You do not indicate the latter which I think is not optimal. Furthermore, all blots should be quantified and presented graphically with the individual data points in the main or supplemental figs or as minimum be summarized in a table. This seems particular important for the data in for instance Fig 6D where IRS1 seems to be higher in some mice and it would be nice to see the quantified data.

o For the western blot data in Fig. 2 please quantify GLUT4 also. Showing more than one muscle would also be nice since these can differ markedly.

o For some bar graphs, you show the individual data points which is very nice. Why not do this for all data?

- o Is muscle mitochondrial protein content and morphology similar?
- o The rationale for using db/db could be improved.
- o The authors should distinguish between chronic “exercise training” vs. acute “exercise bout”

Reviewer #3 (Remarks to the Author):

In the current study, Bian et al. demonstrate that Transgenic mice with an elevation of MG53, a TRIM-family protein member results in longer lifestyle, normal metabolic regulation and increased capacity for wound healing. The experiments are generally well performed and important because they challenge the current dogma that MG53 gain of function, results in insulin resistance. The experiments are generally well performed. However, what is lacking in the current experimental data set is any molecular insight into the mechanism for the enhanced longevity or improved would healing. As written, the MS is very descriptive and provides little with respect to the genes/proteins/pathways that are activated to result in the observed phenotype.

Specific points.

Figure 1 b. Were these data statistically analysed? I am assuming so and this should be indicated.

Figure 2d. Control (littermate control) histologoical cross sections should be included here and the data should be quantified.

Figure 4. The choice of mice running test is a little at odds with what is generally performed which is an endurance test (time to exhaustion at a fixed speed). If there is no fiber type difference, what is responsible for the improved exercise performance? Is it substrate avaiability (eg. Glycogen content of muscles) ond or biogenesis), or something else? These experiments should, be performed.

Reviewer #4 (Remarks to the Author):

Dear Authors,

Your manuscript untitled "Sustained Elevation of MG53 in the Bloodstream Increases Tissue Regenerative Capacity Without Compromising Metabolic Function" is well written and the results obtained are of importance.

The starting hypothesis is based on work showing the regenerative effect of the presence of MG53 in the blood stream of mice. For this article you used two different mouse strains that allow the secretion of MG53 (coupled with tissue plasminogen activator, tPA). The controls used are the WT mouse, the heterozygous mouse or the KO mouse. For my part, I would have carried out a control that allows the expression of inactive MG53 by mutating the Zinc binding sites, for example. Similarly, perhaps a MG53-/- mouse expressing tPA-MG53 could have been a good model for analyzing the role of the secreted MG53.

The presence of this secreted form, in addition to the endogenous form, seems to improve the regeneration of the tissues tested (muscle, skin). Interestingly, tPA-MG53 mice have a better regenerative capacity combined with a better ability to respond to deleterious exercise. These results are generally convincing and are in line with what was found previously. You are concerned about the likely role of MG53 in the development of diabetes and for this reason you have conducted experiments on db/db mice. The results obtained indicate that the presence of tPA-MG53 has no influence on overall metabolic activity.

We would have appreciated to see the effect of MG53 secreted on FAK / ERK activity in addition to IRS1 and PPAR-a to complete the western blot in Figure 6.

Perhaps it would have been wise to test other MG53 partners/substrates. MG53 influences SERCA1 activity, you should test the regulation of calcium entry into the muscle reservoirs of these tPA-MG53 mice compared to WT.

One experiment you could have done was to see if the presence of MG53 in the blood has a direct effect on muscle fiber repair. This could be achieved by crossing these mice with mice deficient in membrane repair protein complex such as the DYSF-/- mice.

In the cardiotoxin experiment, we cannot see the direct role of secreted MG53 nor its presence in the wound site because there is MG53 signal in the muscle cells... In the case of the ear hole healing, we detect the presence of MG53 at and around the lesion. The presence of MG53 in this area may be active (trafficking as you stated) or simply passive with the bleeding associated with the lesion, could you please state this possibility in you text?

In WT mice the healing is much better than in KO, meaning that the muscle responded to this lesion efficiently thanks to the presence of MG53. Finally, the remaining question is how and when in WT mice MG53 is secreted more experiments are needed, probably using the control and model I proposed above.

Some errors and inaccuracies are present in this manuscript which must be corrected before this paper can be accepted.

Here are the minor points :

Figure 1A: Higher-wider bands are present in all mice and visible without knowing which protein it corresponds to. Adding to the same gel the protein extracts from a KO mouse would have eliminated some hypotheses. Why did you choose only one time for WT mice? What is the reason for choosing the age 3 months.

Figure 1B the number of animals tested is missing.

Figure 1C the larger size of the band corresponding to MG53 in skeletal muscle may correspond to either a higher amount of protein or the presence of MG53 not processed, to answer this question a more resolutive migration is warranted.

Figure S2 how the amount (~1-5%) of MG53 in other tissues tested was evaluated?

Figure 2 the left panel in B and C are too close to the right panel the last digit of 150 minutes labels are missing.

For the ITT: in the M&M section it is indicated that blood glucose was assessed at 10,25,45, 90 in the figure 2C one can see data for 120 minutes! Please clarify.

Figure 2E: Those graphs represent a relative quantification (fold of WT), the horizontal black lines in each graphs represent the mean? if it is the mean value, why is this bar not at the level of 1 in the WT?

The movie need a legend indicating the speed of the treadmill, if mice were trained or not and if I understand correctly young mice (-months old) were compared to old tPA-MG53 (32 months).

FigureS4: at which time point the mice were weighed 12 or 16 weeks it is not clear in the legend.

Figure 3A: What is the amount of Rh MG53 deposited on the gel?

Figure 3D: at 14 days post injury the level of healing is quite different thus the presence of fibrosis is also different. It will be of interest to quantify the level of fibrosis at several time points after injury.

Figure 3E what type of statistical test was used?

Figure 4B : the number of animals used is not indicated, it seems that for the WT animals the number is 4 I am not sure this is enough.

Figure 4E: Do you really have data at the 0 m/min speed point? Data for the 6 m/min speed are missing in both mouse groups. I miss the number of animals in this experiment.

Figure 4E : please verify the legend below the graph for statistical significance *: p=0.005 **: P<0.0001 really? In the legend you stated *: P<0.05 **: P<0.01.

Figure 5D: for IgG infiltration and CD11B+ cells, the value captions are incorrect in relation to the graph, please modify accordingly by indicating what the percentage represents? In addition, in the text there is no reference to Figure 5D

Figure 6B : the B label is trimmed.

Figure 6B: In the right panel representing ITT a 120 min value is present.

Figure S5 : number of animals missing.

You stated that you observed nuclear accumulation of MG53 in your tPA-MG53 mice a representative image would be appreciated.

In M&M section serum MG53 quantification please change : The density of thw western blot.

Statistical analysis : Error bars have not always been defined. Nothing have been done to test normality or deviation from it (parametric tests were used). Effect size, statistical power were not evaluated.

Detailed Reponses to Referees (NCOMMS-18-32280)

Reviewer #1

"The authors state that the presence of high levels of circulating MG53 in blood increase tissue regeneration capacity. They test muscle regeneration after cardiotoxin injection and skin regeneration in lesions produced in the ear of the mice. For this purpose they use a transgenic mouse line tPA-MG53 that produces sustained expression of the protein in peripheral blood. The authors state that after 36 months they did not observe side effects on metabolic function. Finally, they claim that sustained overexpression of MG53 in circulation is effective to treat acute muscle tissue injuries and dermal wounds. The paper is interesting and may have therapeutical implications. However there are some weak points.

a. *"I agree that cardiotoxin injury is a common method to assay muscle regeneration in mice but it is far from the muscle injury that is commonly found in patients or in healthy patients suffering sport injuries for instance. I believe that other models such as treadmill exercise would be more realistic*"

Response: We greatly appreciate your suggestion (and the suggestion from other reviewers, see below) for conducting the additional studies evaluating the basal function of mice with treadmill and/or voluntary wheel running exercises. Per your suggestion, we used voluntary wheel running protocol to show that the running capacity of the tPA-MG53 mice is better than the wild type littermates (presented in the new **Fig. 4f**). This finding inspired us to conduct further studies to better understand the myokine function of MG53 in muscle physiology and regeneration.

As suggested by reviewer 2, 3 and 4 (see below), we quantified the glycogen content in muscle fibers (Supplemental **Fig. S8**), measured inflammatory cytokines in the blood (**Fig. S9**), and conducted ultra-structural analysis of mitochondria (**Fig. S10**), all of which did not reveal significant differences between the tPA-MG53 and wild type littermate mice. However, we were excited to find that the Ca signaling machinery in skeletal muscle was significantly improved in the tPA-MG53 mice (see the new **Fig. 4g** and **4h**). This finding is consistent with our previous study that demonstrated a role for MG53 in modulation of store-operated Ca entry in skeletal muscle (ref Ann et al, 2016).

In addition, we provide two exciting new findings in the revised manuscript, per suggestion from reviewer 3 and 4. Specifically, we show that MG53 modulates muscle satellite cell (mSC) proliferation to contribute to muscle regeneration (**Fig. 6**), and rhMG53 protects against eccentric-contraction induced acute and chronic muscle injury in mice (**Fig. 7**). Overall, we thank all reviewers for their insightful suggestions, which have helped to improve our understanding of the role of MG53 in muscle repair and regeneration.

b. *"It would be also interesting to cross the tPA-MG53 with a muscular dystrophy model in a way similar to what the authors did with the db/db mouse to demonstrate a lack of effect in of sustained circulating MG53 on mouse metabolism. Even in acute injury the amount of recombinant MG53 necessary to observe an effect in a hypothetic trial in humans cannot be predicted from the experiments performed since the transgenic models produces a sustained and massive expression of MG53 in plasma".*

Response: While it is possible that crossing the tPA-MG53 mice with other mouse models of muscular dystrophy may provide some information about the safety, efficacy and metabolic function of MG53, the tPA-MG53 mice already provided valuable information about the biology of MG53 in muscle physiology and diabetes: *First*, we know that sustained elevation of MG53 in circulation is safe, since the tPA-MG53 mice lived a healthy life-span (survived past 32 months age). *Second*, we know that MG53 cannot serve as a causative factor for the development of diabetes, since the db/db-tPA-MG53 mice did not show any exacerbated responses to GTT and ITT. *Third*, through immunoblot and ELISA quantification, we found the serum level of MG53 in the tPA-MG53 mice is ~100-fold higher than the wild type littermates, and such level is close to the therapeutic dose of rhMG53 achieved intravenously (1 mg/kg).

Since crossing of these mice will not be feasible for the duration of the time frame, we have conducted an extensive series of studies in collaboration with the Muscle Metabolism Group at the Research and Development Center of GSK. We used eccentric-contraction to induce muscle injury in mice, as an alternative model of chronic muscle injury (to mimic muscular dystrophy). Our findings are very exciting as shown in the new **Fig. 7**. We found that intravenous administration of rhMG53 protects acute muscle injury in a time and dose dependent manner. Even at 6 hours post muscle injury, rhMG53 is still effective. These findings are consistent with a role of MG53 in muscle-injury repair; however, we were very excited to see that repetitive dosing of rhMG53 at 24 hours post

muscle injury still has therapeutic benefits. This unexpected finding provides evidence for a direct role of MG53 in protection against chronic muscle injury which is likely linked to facilitation of muscle satellite cell (mSC) function. Moreover, we found repetitive dosing of rhMG53 is safe and did not alter glucose handling, an observation that was consistent with the healthy lifespan of the tPA-MG53 mice which contained >100-fold elevation of MG53 in their bloodstream.

Reviewer #2

"This paper describes a new mouse model overexpressing MG53 in circulation, which have markedly improved injury repair and regeneration capacity. Overall, I think this paper has some very interesting and novel findings. However, I do have some issues with the manuscript in its present form and I thinks some of the experiments/ measurements lack power or could have been performed in a more optimal way to support the conclusions:

Response: We appreciate the reviewer's endorsement of the novelty and the significance of our study testing the safety and efficacy with sustained elevation of MG53 in maintaining muscle health and regeneration following injury. We thank the reviewer for his/her many constructive comments and suggestions to improve the clarity and statistics of our study in testing the myokine function of MG53 in tissue repair and regeneration. We have followed the recommendations of all four reviewers conducted additional experiments requested by you and the other reviewers. Our point-to-point responses are provided below.

<u>*Major:*</u>

a. "*For Fig 2C+D, there seems to be a tendency towards increased insulin action in the MG53 mice and the error-bars are huge with n=5-6. Suggest to add more mice to show convincingly whether the difference is there or not. If different, the reason for the difference should be ascertained. Is insulin-signaling increased in different metabolic tissues? Is glucose uptake? Do the mice then secrete less insulin during the GTT to explain the lack of difference in GTT?*"

Response: Our available data did not reveal statistical differences between the wild type and tPA-MG53 mice in terms of ITT and GTT. Because of the large standard deviations with the ITT measurements in **Fig. 2c**, we have conducted additional ITT with another group of mice at the age of 12 weeks (n=6 for wild type; n=6 for tPA-MG53), and found no statistical differences between the two groups of mice. These data are provided as supplemental **Fig. S6**.

Regarding insulin signaling in the tPA-MG53 muscle, we have performed additional biochemical studies to test the response of skeletal muscle to insulin stimulation. Please see our response in <u>***d***</u>, and new text on page 5 (line 150-158).

b. *"For the running performance data in Fig 4E, the interpretation is that the recovery from prior exercise is improved in MG53 mice. Was the running performance in response to a single bout of acute exercise the same or is this phenotype only seen with repeated bouts, i.e. is there a difference in base-line exercise capacity? Is the phenotype muscle-injury related (inflammatory markers, myopathy markers, injury markers) or is this a metabolic effect (mitochondrial proteins, glucose and fat metabolism protein markers)? Is this also a phenotype seen with less extreme exercise regimens? Is exercise-induced substrate utilization changed? At the very least, I suggest to quantify the acute exercise capacity of these mice using a conventional incremental ramp test and perform some of the same analyses as in Fig 5 at the time where you observe the reduced running capacity. The n in this experiment is not stated in the legend".*

Response: These points were also raised by reviewer #1 and #3. Please see our response above. The n number for the individual studies are present in the figure legend. To address your specific suggestions, we have included the following paragraphs to our revised manuscript (page 6-7, line 210-229):

> To evaluate if sustained elevation of MG53 affects the basal performance of mice, the mice were subjected to voluntary wheel running for 7 days. As shown in **Fig. 4f**, the tPA-MG53 mice showed enhanced running capacity than that of wild type littermate controls (n=5 per group). This enhanced muscle performance did not reflect changes in glycogen content of the muscle fiber, since biochemical studies revealed similar level of glycogen in the gastrocnemic muscle derived from both wild type and tPA-MG53 littermates (see Supplemental **Fig. S8**). We measured inflammatory cytokines in blood before and after voluntary wheel running, and did not observe any difference in the levels of cytokines between tPA-MG53 and wild type littermates (**Fig. S9**). Moreover, electron microscopy analysis did not show measurable changes in

mitochondria content in both EDL and soleus muscles derived from the tPA-MG53 mice compared with wild type littermates (see Supplemental **Fig. S10**).

Previously we reported that MG53 plays a role in modulation of Ca signaling in skeletal muscle[34]. To test if sustained elevation of MG53 impacts Ca signaling in skeletal muscle, we isolated flexor digitorium brevis (FDB) muscle from wild type and tPA-MG53 littermates. As shown in **Fig. S11**, KCl-induced intracellular Ca release is comparable between wild type and tPA-MG53 muscle fibers in the absence of extracellular Ca. Interestingly, with 2 mM Ca present in the extracellular solution, sustained Ca release was observed in muscle fiber derived from the tPA-MG53 mice (**Fig. 4g**). The half-time for Ca decay was 45.1±9.0s (tPA-MG53), and 15.2±4.4s (wild type, $p<0.05$) (**Fig. 4h**), suggesting that increased MG53 expression could enhance extracellular Ca entry in skeletal muscle[34]. This may be a contributing factor for the enhanced muscle performance of the tPA-MG53 mice. Further studies will be needed to ascertain the biological base of this observation.

c. *You state in the abstract that the MG53 mice live a healthier and longer life-span. This seems to be based on the observation in the results that 3 of 5 mice survived over 36 months whereas all WT mice died below 30 mo. +How many mice in the WT group and was this significant? Technically, I acknowledge that the statement regarding life-span is correct but n=5 seems a bit low for life-span analysis (or any other kind). In terms of health, you seem to refer to Fig 1D and S3 as evidence for a healthy life span with no observable pathology. However, there seem to be no control mice quantified making it very difficult to judge how healthy the phenotype looks. Also, how many mice are the shown stainings representative of?*

Response: We appreciate the reviewer's comments regarding the life-span study with the tPA-MG53 mice. Clearly, more animals and additional analysis will be required to arrive at the mechanistic base for the impact of MG53 elevation on longevity. To alleviate these concerns, we have revised abstract accordingly: "Transgenic mice with sustained elevation of MG53 in the bloodstream (tPA-MG53) lived a healthy~~ier and longer~~ life-span ~~when compared with littermate wild type mice~~". We stated in the legend of **Fig. 1d**: The pictures are representative of 2 other tPA-MG53 mice at 32 months age.

For the histological analysis of the different vital organs, we have added H/E staining results from additional wild type and tPA-MG53 mice at the age of 24 months (see **Fig. S4**).

d. *You state that the MG53 mice have normal insulin signalling. This seems to refer to the total protein blots in Fig 2. You should run a +/- insulin-stimulation setup, e.g. your ITT, take tissues and measure some insulin-responsive phosphorylations or modify these statement.*

Response: We have followed the reviewer's suggestion to challenge mice with insulin and performed western blot analysis with the fresh muscle. The data shown in **Fig. 2f** demonstrated no significant differences in activation of key insulin pathway proteins between tPA-MG53 and wild type controls with insulin treatment.

<u>Minor</u>

e. *Representative western blots provide 2 important pieces of information to the reader. 1) Is the signal/noise ratio reasonable 2) Does the band run at the correct MW. You do not indicate the latter which I think is not optimal. Furthermore, all blots should be quantified and presented graphically with the individual data points in the main or supplemental figs or as minimum be summarized in a table. This seems particular important for the data in for instance Fig 6D where IRS1 seems to be higher in some mice and it would be nice to see the quantified data.*

Response: We will include full Western blot film pictures with molecular weight markers upon acceptance of the manuscript. Moreover, we used dot plot for quantification of the intensity of the protein band in all western blots. These new figures are **Fig. 2e** and **Fig. S7** in the revised manuscript.

f. *For the western blot data in Fig. 2 please quantify GLUT4 also. Showing more than one muscle would also be nice since these can differ markedly. For some bar graphs, you show the individual data points which is very nice. Why not do this for all data?*

Response: The quantification of GLUT4 is now provided in **Fig. 2e**. As suggested, we used dot plot to show the individual data points for all western blots in the manuscript.

g. *Is muscle mitochondrial protein content and morphology similar?*

Response: This is an excellent question. We have done two additional studies. As recommended by Reviewer 3, we did the quantification of glycogen content in skeletal muscle derived from the wild type and tPA-MG53 littermates (see supplemental **Fig. S8**, and response to Reviewer 3). There appears to be a trend for higher glycogen content in the tPA-MG53 muscle compared with the wild type muscle, but the p value does not support statistical significance (p=0.2398). We also used transmission electron microscopy analysis to resolve the mitochondria content and morphology. The data shown in Supplemental **Fig. S10** show that the tPA-MG53 muscles maintain normal mitochondrial structure and network.

h. *The authors should distinguish between chronic "exercise training" vs. acute "exercise bout"*

Response: Agree. We have made appropriate changes in the revised text.

i. *The rationale for using db/db could be improved.*

Response: Since our initial discovery of MG53 in 2009, many researchers have joined our team effort in exploring the physiology and the potential of targeting MG53-mediated tissue repair in regenerative medicine. A study published by researchers from Peking University reported an adverse effect of MG53 as an E3-ligase in harnessing IRS1-mediated insulin signaling and metabolic function in muscle (Refs 20, 30, 38). Specifically, they reported that muscle samples derived from the *db/db* mice and human patients show elevation of MG53 protein. This result has not been reproduced by any other laboratories in the community (Refs 16, 21, 22, 23). Moreover, their proposed role for MG53 down-regulation of IRS-1 protein expression as a causative factor for the development of type II diabetes lacks biological base, since previous studies (Refs 24, 25) demonstrated that genetic ablation of IRS-1 did not lead to type II diabetic phenotype in mice.

More recently, the same group from Peking University published another paper in Circulation stating that an immuno-approach using antibody against MG53 to reduce serum level of MG53 in the blood can have potential to reduce blood glucose levels in diabetes (Wu et al, 2019). This paper has many controversies as stated in our letter to the editor. Please see the attached PDF file which has been accepted for publication by Circulation.

We have added this new information to our discussion in the revised manuscript (see page 10, line 378-389). The rationale for the use of *db/db* mice is now clearly stated in the revised Introduction. Please see page 3, line 67-73.

Reviewer #3

"*In the current study, Bian et al. demonstrate that transgenic mice with an elevation of MG53, a TRIM-family protein member results in longer lifestyle, normal metabolic regulation and increased capacity for wound healing. The experiments are generally well performed and important because they challenge the current dogma that MG53 gain of function, results in insulin resistance. The experiments are generally well performed. However, what is lacking in the current experimental data set is any molecular insight into the mechanism for the enhanced longevity or improved would healing. As written, the MS is very descriptive and provides little with respect to the genes/proteins/pathways that are activated to result in the observed phenotype*".

Response: We appreciate the reviewer's suggestion that more studies are required to arrive at the "*molecular insight into the mechanism for the enhanced longevity or improved would healing",* and further testing is needed to ascertain the direct action of circulating MG53 that underlies *the observed phenotype* of improved muscle regeneration with elevation of MG53 protein in the bloodstream

In the revised manuscript, we provide two new findings that provide insight into the role of MG53 in facilitating wound healing and muscle regeneration, as well as the direct action of circulating MG53 in protecting against chronic muscle injury.

In the new **Fig. 6**, we show that MG53 modulates muscle satellite cell (mSC) proliferation to contribute to muscle regeneration. One main finding of our study is that sustained elevation of MG53 in circulation leads to increased regenerative capacity of skeletal muscle associated with cardiotoxin (**Fig. 5**) and exercise-induced muscle injury (**Fig. 4**). We isolated mSCs from *mg53-/-*, wild type, and tPA-MG53 mice, and found defective mSC proliferation from *mg53-/-* muscle and significantly improved mSC proliferation in tPA-MG53 muscle, compared with mSC derived from wild type muscle. More importantly, we found that incubation of *mg53-/-* muscle fibers with rhMG53 protein could restore mSC proliferation. Identification of a role of MG53 in modulation of mSC function provides a mechanistic base for the increased muscle regenerative capacity associated with injury.

To gain further insight into the direct action of circulating MG53 in protection against chronic muscle injury, we conducted an extensive series of studies using eccentric-contraction to induce muscle injury in mice (**Fig. 7**). We found that intravenous administration of rhMG53 protects acute muscle injury in a time and dose dependent manner. Even at 6 hours post muscle injury, rhMG53 is still effective. These findings are consistent with a role of MG53 in muscle-injury repair; however, we were very excited to see that repetitive dosing of rhMG53 at 24 hours post muscle injury still has therapeutic benefits. This unexpected finding provides evidence for a direct role of MG53 in protection against chronic muscle injury which is likely linked to facilitation of mSC function. Moreover, we found repetitive dosing of rhMG53 is safe and did not alter glucose handling, an observation that was consistent with the healthy lifespan of the tPA-MG53 mice which contained >100-fold elevation of MG53 in their bloodstream.

In addition to the new data shown in **Fig. 6** and **7**, we have followed the suggestions by all four reviewers and conducted the additional experiments as recommended, which all advance our understanding of the biological, physiological and potential translational values of MG53 in muscle physiology and disease. Specifically, data presented in **Fig. 1c, 2e, 2f, 4b, 4f, 4g,** and **4h** are new to the revised manuscript. Moreover, we included 9 additional figures to the Supplemental Materials: **Fig. S1, S4, S6, S7, S8, S9, S10, S11, S13.**

Overall, we are grateful to all reviewers for the insightful suggestion, which have certainly helped to improve our understanding of the role of MG53 in muscle physiology.

<u>Specific points</u>

a. *Figure 1 b. Were these data statistically analysed? I am assuming so and this should be indicated.*

Response: The difference in basal level of MG53 in sera derived from wild type and tPA-MG53 mice are significantly different with p value < 0.0001. This has been added to the figure legend.

b. *Figure 1d. Control (littermate control) histological cross sections should be included here and the data should be quantified.*

Response: We apologize for not having the littermate control for the wild type mice in the histological analysis in the original manuscript. Per your suggestion and that of reviewer #2, we have included the H/E analysis with wild type and tPA-MG53 mice at the age of 24 months, in the revised Supplemental **Fig. S4**. The main point of **Fig. 1d** is to show that no gross anatomical abnormality was present in the aged tPA-MG53 mice (32 months old). The H/E pictures shown in **Fig. 1d** with the different vital organs are representative of 3 other mice at the age of 32 months.

c. *Figure 4. The choice of mice running test is a little at odds with what is generally performed which is an endurance test (time to exhaustion at a fixed speed). If there is no fiber type difference, what is responsible for the improved exercise performance? Is it substrate availability (eg. Glycogen content of muscles) ond or biogenesis), or something else? These experiments should, be performed.*

Response: We greatly appreciate this recommendation along with reviewers #1 and #2, for their insightful suggestions. As stated in our response to reviewer #1 (***<u>a</u>*** <u>and</u> ***<u>b</u>***) and #2 (***<u>b</u>***), fulfillment of the additional experiments recommended by you all has enhanced our understanding on the role of MG53 in muscle physiology and regeneration. We have obtained exciting data which shows that skeletal muscle derived from the tPA-MG53 muscle displayed enhanced extracellular Ca entry (**Fig. 4g, 4h**, which is consistent with our published data, Ann et al, 2016) which may be a contributing factor for the enhanced muscle performance. Future studies will be required to dissect the mechanistic base that underlies the role of MG53 in modulation of store-operated Ca entry.

Reviewer #4

a. *"Your manuscript untitled "Sustained Elevation of MG53 in the Bloodstream Increases Tissue Regenerative Capacity Without Compromising Metabolic Function" is well written and the results obtained are of importance. The starting hypothesis is based on work showing the regenerative effect of the presence of MG53 in the blood stream of mice. For this article you used two different mouse strains that allow the secretion of MG53 (coupled with tissue plasminogen activator, tPA). The controls used are the WT mouse, the heterozygous mouse or the KO mouse. For my part, I would have carried out a control that allows the expression of inactive MG53 by mutating the Zinc binding sites, for example. Similarly, perhaps a MG53-/- mouse expressing tPA-MG53 could have been a good model for analyzing the role of the secreted MG53".*

Response: This is an excellent suggestion. While generation of transgenic mice carrying a mutant MG53 with disrupted Zinc binding capacity, or crossing of the tPA-MG53 mice with the *mg53-/-* mice, may provide further insight into the role of circulating MG53 in muscle repair and regeneration, we have conducted an extensive series of study with the Muscle Metabolism Group at the R&D Center of GSK, using an alternative model of eccentric-contraction induced chronic muscle injury in mice. Our findings are very exciting as shown in the new **Fig. 7**. We found that intravenous administration of rhMG53 protects acute muscle injury in a time and dose dependent manner. Even at 6 hours post muscle injury, rhMG53 is still effective. These findings are consistent with a role of MG53 in muscle-injury repair; however, we were very excited to see that repetitive dosing of rhMG53 at 24 hours post muscle injury still has therapeutic benefits. This unexpected finding provides evidence for a direct role of MG53 in protection against chronic muscle injury which is likely linked to facilitation of mSC function. Moreover, we found repetitive dosing of rhMG53 is safe and did not alter glucose handling, an observation that was consistent with the healthy lifespan of the tPA-MG53 mice which contained >100-fold elevation of MG53 in their bloodstream.

Thank you for your suggestion. In the future, we will consider creating transgenic mice that carry a dominant negative mutation of MG53, which can be used to test the intermolecular interaction between MG53 and its interacting partners in the physiological function of muscle injury repair response.

b. *"The presence of this secreted form, in addition to the endogenous form, seems to improve the regeneration of the tissues tested (muscle, skin). Interestingly, tPA-MG53 mice have a better regenerative capacity combined with a better ability to respond to deleterious exercise. These results are generally convincing and are in line with what was found previously. You are concerned about the likely role of MG53 in the development of diabetes and for this reason you have conducted experiments on db/db mice. The results obtained indicate that the presence of tPA-MG53 has no influence on overall metabolic activity. We would have appreciated to see the effect of MG53 secreted on FAK / ERK activity in addition to IRS1 and PPAR-a to complete the western blot in Figure 6".*

Response: We have now provided additional western blot with quantification of FAK and ERK in muscle samples derived from the wild type and tPA-MG53 mice. Please see supplemental **Fig. S7**.

c. *"Perhaps it would have been wise to test other MG53 partners/substrates. MG53 influences SERCA1 activity, you should test the regulation of calcium entry into the muscle reservoirs of these tPA-MG53 mice compared to WT"*

Response: This is an excellent point. We have conducted Ca measurement in FDB fibers derived from the wild type and tPA-MG53 littermates. These results are presented in **Fig. 4g, 4h**, and Supplemental **Fig. S11**. We found that KCl-induced intracellular Ca release is similar between wild type and tPA-MG53 mice when the experiments were performed with zero Ca present in the extracellular solution (**Fig. S11**). However, with 2 mM present in the extracellular solution, the KCl-induced Calcium release show a sustained phase in tPA-MG53 muscle fibers when compared with the wild type muscle fiber (**Fig. 4g and 4h**). These findings are consistent with our published study demonstrating the role of MG53 in regulation of extracellular Ca entry (Ann et al, 2016).

d. *"One experiment you could have done was to see if the presence of MG53 in the blood has a direct effect on muscle fiber repair. This could be achieved by crossing these mice with mice deficient in membrane repair protein complex such as the DYSF-/- mice".*

Response: This is an excellent suggestion. A recent publication from an independent group has shown that rhMG53 treatment could improve muscle function in *dysferlin-/-* mice (ref 36). We now acknowledge this publication in the revised discussion.

e. *"In the cardiotoxin experiment, we cannot see the direct role of secreted MG53 nor its presence in the wound site because there is MG53 signal in the muscle cells... In the case of the ear hole healing, we detect the presence of MG53 at and around the lesion. The presence of MG53 in this area may be active (trafficking as you stated) or simply passive with the bleeding associated with the lesion, could you please state this possibility in you text?* In WT mice the healing is much better than in KO, meaning that the muscle responded to this lesion efficiently thanks to the presence of MG53. Finally, the remaining question is how and when in WT mice MG53 is secreted more experiments are needed, probably using the control and model I proposed above"

Response: We appreciate your thoughtful suggestions and have revised our manuscript to take into account your comments. Please see page 6, line 178-179 in the revised text. For the secretion of MG53 in response to injury, our previous publication has done some pilot studies (*J Mol Cell Cardiol*. 2015 Mar;80:10-19. doi: 10.1016/j.yjmcc.2014.12.010.). We have also included some more discussion about secretion of MG53 for injury repair in the revised discussion section.

f. *"Some errors and inaccuracies are present in this manuscript which must be corrected before this paper can be accepted".*

Response: Thank you, we corrected the errors in the revised manuscript.

Minor points

a. *Figure 1A: Higher-wider bands are present in all mice and visible without knowing which protein it corresponds to. Adding to the same gel the protein extracts from a KO mouse would have eliminated some hypotheses. Why did you choose only one time for WT mice? What is the reason for choosing the age 3 months. Figure 1B the number of animals tested is missing.*

Response: The higher bands with western blot of the serum samples are Albumin. We have now labeled the MG53 band in the western blot picture. The western blot pictures presented are representative of mice at all ages spanning from 2 months to 32 months. As recommended, we provided a new supplemental **Fig. S1** to show the specificity of the antibody used where serum derived from the *mg53-/-* mice was used as a negative control. The number of animals has been added to **Fig 1b**.

b. *Figure 1C the larger size of the band corresponding to MG53 in skeletal muscle may correspond to either a higher amount of protein or the presence of MG53 not processed, to answer this question a more resolutive migration is warranted.*

Response: The larger size of the MG53 band detected in western blot of the tPA-MG53 muscle represents the doublet pattern of the native 53 kD MG53 plus the unprocessed tPA-MG53 protein (due the addition of the tPA signal peptide, 21 amino acid).

c. *Figure S2 how the amount (~1-5%) of MG53 in other tissues tested was evaluated?*

Response: The lower level MG53 protein present in non-muscle tissues has been reported in our previous publications (*Nat Commun.* 2014; 5: 4387. doi: 10.1038/ncomms5387; *Sci Transl Med.* 2015 7(279): 279ra36. doi: 10.1126/scitranslmed.3010755). To take this into consideration, we loaded 20 µg protein for the kidney and lung tissue, whereas for the muscle we used 2 µg for the western blot.

d. *Figure 2 the left panel in B and C are too close to the right panel the last digit of 150 minutes labels are missing.*

Response: This figure has been modified.

e. *For the ITT: in the M&M section it is indicated that blood glucose was assessed at 10,25,45, 90 in the figure 2C one can see data for 120 minutes! Please clarify.*

Response: We apologize for our oversight, and have added the 120 minute time point in the Materials and Methods section, see page 14, line 465.

f. *Figure 2E: Those graphs represent a relative quantification (fold of WT), the horizontal black lines in each graphs represent the mean? if it is the mean value, why is this bar not at the level of 1 in the WT?*

Response: We routinely used multiple samples derived from the wild type and tPA-MG53 samples in a given western blot. To illustrate the spread of the band intensity with the wild type samples, we normalize the band intensity to one wild type sample (which is often the one with middle intensity), and plotted the relative intensity of other samples (including wild type and tPA-MG53 muscles) in the scatter plot. These details have been added to the revised MM section, see page 15, line 520-523.

g. *The movie need a legend indicating the speed of the treadmill, if mice were trained or not and if I understand correctly young mice (-months old) were compared to old tPA-MG53 (32 months).*

Response: The mice have been trained on the treadmill for 2 hours. The treadmill was running at 10 meter/minutes. This legend has been added to the supplemental movies. Yes, the movie was taken with young wild type mice at 6 months age and aged tPA-MG53 mice at 32 months age.

h. *FigureS4: at which time point the mice were weighed 12 or 16 weeks it is not clear in the legend.*

Response: We have added this information to the legend for **Fig. S5**. More specifically, body weight was assessed between 11 to 15 weeks of age. A panel depicting the average age at body weight measurement for each genotype has also been included in **Fig. S5**.

i. *Figure 3A: What is the amount of Rh MG53 deposited on the gel?*

Response: The amount of rhMG53 as a reference standard is provided in the Figure legend, see page18, line 714.

j. *Figure 3D: at 14 days post injury the level of healing is quite different thus the presence of fibrosis is also different. It will be of interest to quantify the level of fibrosis at several time points after injury.*

Response: The role of MG53 in controlling tissue fibrosis following injury has been published previously in JBC (Li et al). Therefore, we only conducted quantification of fibrosis at the end stage experiment.

k. *Figure 3E what type of statistical test was used?*

Response: The method used for statistical analysis is now specified in the Materials and Methods section.

l. *Figure 4B: the number of animals used is not indicated, it seems that for the WT animals the number is 4 I am not sure this is enough.*

Response: We have now included more quantification of muscle weight in the revised **Fig. 4b**.

m. *Figure 4E: Do you really have data at the 0 m/min speed point? Data for the 6 m/min speed are missing in both mouse groups. I miss the number of animals in this experiment.*

Response: The x-axis in **Fig. 4e** ranges from 6 m/min to 16 m/min. The first data point is 6 m/min, which was mistakenly labelled as 0 m/min. We have corrected this labeling in the new **Fig. 4e**. We performed the test with mice running at 6 m/min speed.

n. *Figure 4E: please verify the legend below the graph for statistical significance *: p=0.005 **: P<0.0001 really? In the legend you stated *: P<0.05 **: P<0.01.*

Response: The accurate p-value has been added to this figure legend. We apologize for this confusion.

o. *Figure 5D: for IgG infiltration and CD11B+ cells, the value captions are incorrect in relation to the graph, please modify accordingly by indicating what the percentage represents? In addition, in the text there is no reference to Figure 5D*

Response: For quantification of immune cell filtration following cardiotoxin injury, we counted the actual number of CD11b positive cells near the region of muscle injury. Thus, we have corrected the figure legend accordingly (Page 22, line 806-807). Thank you for pointing out this error. We have also added the text in the revised Result section that refers to the muscle fiber measurement.

p. *Figure 6B: the B label is trimmed.*

Response: The panel has been corrected.

q. Figure 6B: In the right panel representing ITT a 120 min value is present.

Response: We have included the 120 minute time point in the revised methods section.

r. Figure S5: number of animals missing.

Response: The number has been added to the figure legend.

s. You stated that you observed nuclear accumulation of MG53 in your tPA-MG53 mice a representative image would be appreciated.

Response: A picture showing nuclear localization of MG53 in heart muscle derived from the tPA-MG53 mice is now presented in supplemental data, see **Fig. S13**.

t. In M&M section serum MG53 quantification please change: The density of thw western blot.

Response: The typo has been corrected in the revised manuscript.

u. Statistical analysis: Error bars have not always been defined. Nothing have been done to test normality or deviation from it (parametric tests were used). Effect size, statistical power were not evaluated.

Response: In the revised figures, we have presented data uniformly in the format of mean±s.d, following the recommendation for most journals. We also revised the Statistical Analysis in the method section to state the parametric tests used to evaluate normality of the data.

REVIEWERS' COMMENTS:

Reviewer #1 (Remarks to the Author):

The authors have addressed my comments satisfactorily.

Reviewer #2 (Remarks to the Author):

In general, this reviewer is satisfied with the revision.

Only minor issue to consider is the following statement from the rebuttal with a similar wording in the main text:

As shown in Fig. 4f, the tPA-MG53 mice showed enhanced running capacity than that of wild type littermate controls (n=5 per group). This enhanced muscle performance did not reflect changes in glycogen content of the muscle fiber, since biochemical studies revealed similar level of glycogen in the gastrocnemic muscle derived from both wild type and tPA-MG53 littermates (see Supplemental Fig. S8).

The difference in voluntary running capacity is interpreted as relating to "enhanced muscle performance". However, the phenotype might relate to something other than muscle performance, e.g. motivation to run. Is there a difference in running performance in response to forced treadmill running (max running duration or intensity)? If not, this might argue that it is not "enhanced muscle performance".

To conclude regarding muscle-specific performance, you must show that the isolated or artifically stimulated muscle performs better (e.g. electrical stimulation in vitro/in situ with force and fatigue-measurements).

I suggest as a minimum to rephrase the data interepretation in a way, where you present "enhanced muscle performance" as one of several possibilities/explanations.

Reviewer #3 (Remarks to the Author):

The authors have done a fine job addressing my concerns. The manuscript is much stronger now.

Reviewer #4 (Remarks to the Author):

Thank you for the work you have done to respond to all my comments. The manuscript is now improved. The various errors and inadequacies have been corrected.

Dr Marc Bartoli

<u>Reviewer #2 (Comments to the Author)</u>

In general, this reviewer is satisfied with the revision. Only minor issue to consider is the following statement from the rebuttal with a similar wording in the main text:

As shown in Fig. 4f, the tPA-MG53 mice showed enhanced running capacity than that of wild type littermate controls (n=5 per group). This enhanced muscle performance did not reflect changes in glycogen content of the muscle fiber, since biochemical studies revealed similar level of glycogen in the gastrocnemic muscle derived from both wild type and tPA-MG53 littermates (see Supplemental Fig. S8).

The difference in voluntary running capacity is interpreted as relating to "enhanced muscle performance". However, the phenotype might relate to something other than muscle performance, e.g. motivation to run. Is there a difference in running performance in response to forced treadmill running (max running duration or intensity)? If not, this might argue that it is not "enhanced muscle performance".

To conclude regarding muscle-specific performance, you must show that the isolated or artifically stimulated muscle performs better (e.g. electrical stimulation in vitro/in situ with force and fatigue-measurements).

I suggest as a minimum to rephrase the data interepretation in a way, where you present "enhanced muscle performance" as one of several possibilities/explanations.

<u>Response:</u>

We completely agree with the reviewer's suggestion to "rephrase the data interpretation in a way, where you present "enhanced muscle performance" as one of several possibilities/explanations". Accordingly, we have removed the phrase "this enhanced muscle performance" from the text. The revised text now reads as follows (see line 226-227, in the revised manuscript with tracking changes):

"As shown in Fig. 4f, the tPA-MG53 mice showed enhanced running capacity compared to wild type littermate controls (n=5 per group), which did not reflect changes in glycogen content of the muscle fiber since biochemical studies revealed only marginal increase in glycogen content in gastrocnemic muscle derived from tPA-MG53 mice compared with wild type littermates".
